# Revealing Tissue Heterogeneity and Spatial Dark Genes from Spatially Resolved Transcriptomics by Multiview Graph Networks

**DOI:** 10.34133/research.0228

**Published:** 2023-09-20

**Authors:** Ying Li, Yuejing Lu, Chen Kang, Peiluan Li, Luonan Chen

**Affiliations:** ^1^ School of Mathematics and Statistics, Henan University of Science and Technology, Luoyang, 471023, China.; ^2^ Longmen Laboratory, Luoyang, Henan, 471003, China.; ^3^ Key Laboratory of Systems Biology, Institute of Biochemistry and Cell Biology, Center for Excellence in Molecular Cell Science, Chinese Academy of Sciences, Shanghai, 201100, China.; ^4^ Key Laboratory of Systems Health Science of Zhejiang Province, Hangzhou Institute for Advanced Study, University of Chinese Academy of Sciences, Hangzhou, 310000, China.; ^5^ School of Life Science and Technology, ShanghaiTech University, Shanghai, 201100, China.

## Abstract

Spatially resolved transcriptomics (SRT) is capable of comprehensively characterizing gene expression patterns and providing an unbiased image of spatial composition. To fully understand the organizational complexity and tumor immune escape mechanism, we propose stMGATF, a multiview graph attention fusion model that integrates gene expression, histological images, spatial location, and gene association. To better extract information, stMGATF exploits SimCLRv2 for visual feature exaction and employs edge feature enhanced graph attention networks for the learning potential embedding of each view. A global attention mechanism is used to adaptively integrate 3 views to obtain low-dimensional representation. Applied to diverse SRT datasets, stMGATF is robust and outperforms other methods in detecting spatial domains and denoising data even with different resolutions and platforms. In particular, stMGATF contributes to the elucidation of tissue heterogeneity and extraction of 3-dimensional expression domains. Importantly, considering the associations between genes in tumors, stMGATF can identify the spatial dark genes ignored by traditional methods, which can be used to predict tumor-driving transcription factors and reveal tumor immune escape mechanisms, providing theoretical evidence for the development of new immunotherapeutic strategies.

## Introduction

Spatially resolved transcriptomics (SRT) can obtain information on the spatial locations of genes within tissues while detecting cellular gene expression levels, making substantial contributions such as in tumor heterogeneity and immunity. SRT technologies include in situ capture and sequencing-based technologies such as 10x Genomics Visium and Slide-seq and image-based technologies such as STARmap, which have a important role in discovering new biomarkers, dissecting tumor heterogeneity, and identifying therapeutic responses [1].

Several algorithms have been designed to predict the spatial distribution of cell types and gene expressions. SpatialDE identifies genes with expression changes in spatial patterns from high-throughput spatial resolution gene expression data [2]. stLearn has integrated analysis methods that use all 3 types of data—gene expression, spatial information, and tissue morphology—to discover cell types and reconstruct cell type evolution [3]. Giotto implements algorithms to describe tissue composition, spatial expression patterns, and cell–cell interactions [4]. BayesSpace uses spatial neighborhood information to enhance the resolution of SRT data and perform clustering analysis [5]. SEDR is an unsupervised method that uses a deep autoencoder to construct a low-dimensional latent representation of gene expression [6]. SpaGCN is a graph convolutional network method for analyzing SRT data, which aggregates gene expression data from neighboring spots [7]. STAGATE is a graph attention autoencoder framework that can accurately identify spatial domains in SRT data by learning to merge low-dimensional latent embeddings of spatial information and gene expression profiles [8]. stMVC is a multiview graph collaborative learning model, which integrates organoid, gene expression, spatial location, and biological background through a graph attention network (GAT) to better understand tissue heterogeneity [9].

While these methods have many interesting findings, there are limitations such as spatial location information and edge feature exploitation, and robust fusion representation extraction. Specifically, although stMVC can adaptively learn node features and graph topology using a GAT, it does not consider multidimensional edge information, which makes fusion information less effective. In addition, it uses a semisupervised attention mechanism to collaboratively learn the fused features of both views but cannot capture the important features of the channel, spatial width, and spatial height to better learn a robust representation of the integrated features.

While some methods take spatial information into account, they ignore the association between genes, which can provide more information about biological processes such as transcriptional regulation. Nor do some take into account the batch effect of adjacent slices; hence, they cannot be applied to multiple contiguous sections to profile gene expression patterns in a 3-dimensional (3D) context, such as to reconstruct 3D tissue models and extract 3D expression domains.

To address these issues, we propose a multiview graph attention fusion framework (stMGATF) that can synthesize information such as gene expression, histological images, spatial location, and gene association to elucidate tissue heterogeneity and identify spatial dark genes (SDGs) that may play an important role in disease progression. stMGATF extracts visual features of each spot by SimCLRv2, which learns better representations after fine-tuning in downstream tasks. Edge feature enhanced GAT (EGAT) obtains the potential embedding of each view of histological similarity graphs (HSGs), spatial location graphs (SLGs), and gene association graphs (GAGs). EGAT facilitates the efficient use of multidimensional real-valued edge information by making 1-dimensional edge features adaptive across layers, thus enabling more effective fusion of information in node features and graph topology. The 3 views are adaptively integrated to obtain low-dimensional representation using a global attention mechanism (GAM), which improves the performance of neural networks even with different structures and depths, featuring data scaling and robustness, and introducing 3D permutation with a multilayer perceptron for channel attention alongside a convolutional spatial attention submodule. Moreover, the robustness of the stMGATF is validated by hyperparametric analysis and 10-fold cross-validation.

stMGATF outperforms existing methods in denoising original data and identifying spatial domains with coherent expression and histology, especially in detecting more domains enriched for cancer regions. It contributes to the elucidation of tissue heterogeneity in identifying cell states and distinct spatial regions of tumor margins and tumor cells associated with cell proliferation and migration. stMGATF is conducive to the reconstruction of 3D tissue models to identify 3D expression domains, using multidimensional edge information by processing neighboring slice information. Moreover, it reveals the layer-specific marker genes on a mouse visual cortex dataset profiled by STARmap, indicating that stMGATF can process data even with different resolutions. Importantly, considering the associations between genes, stMGATF reconstructs the spatial pseudo-expression (SPE), based on which it can identify the SDGs ignored by traditional methods, which do not differ significantly in terms of gene expression but differ significantly in terms of SPE. The SDGs identified by stMGATF have the potential to play a role in tumor development, including its effects on cell proliferation, invasion, and metastasis. SDGs are closely correlated with tumor progression and can effectively predict the prognosis of tumors and their driving transcription factors (TFs). Specifically, the hub upstream TF *ZNF469* predicted by SDGs promotes immune escape to drive tumor growth and can serve as a potential immunotherapeutic target.

## Results

### Overview of the stMGATF framework

**Fig. 1.**
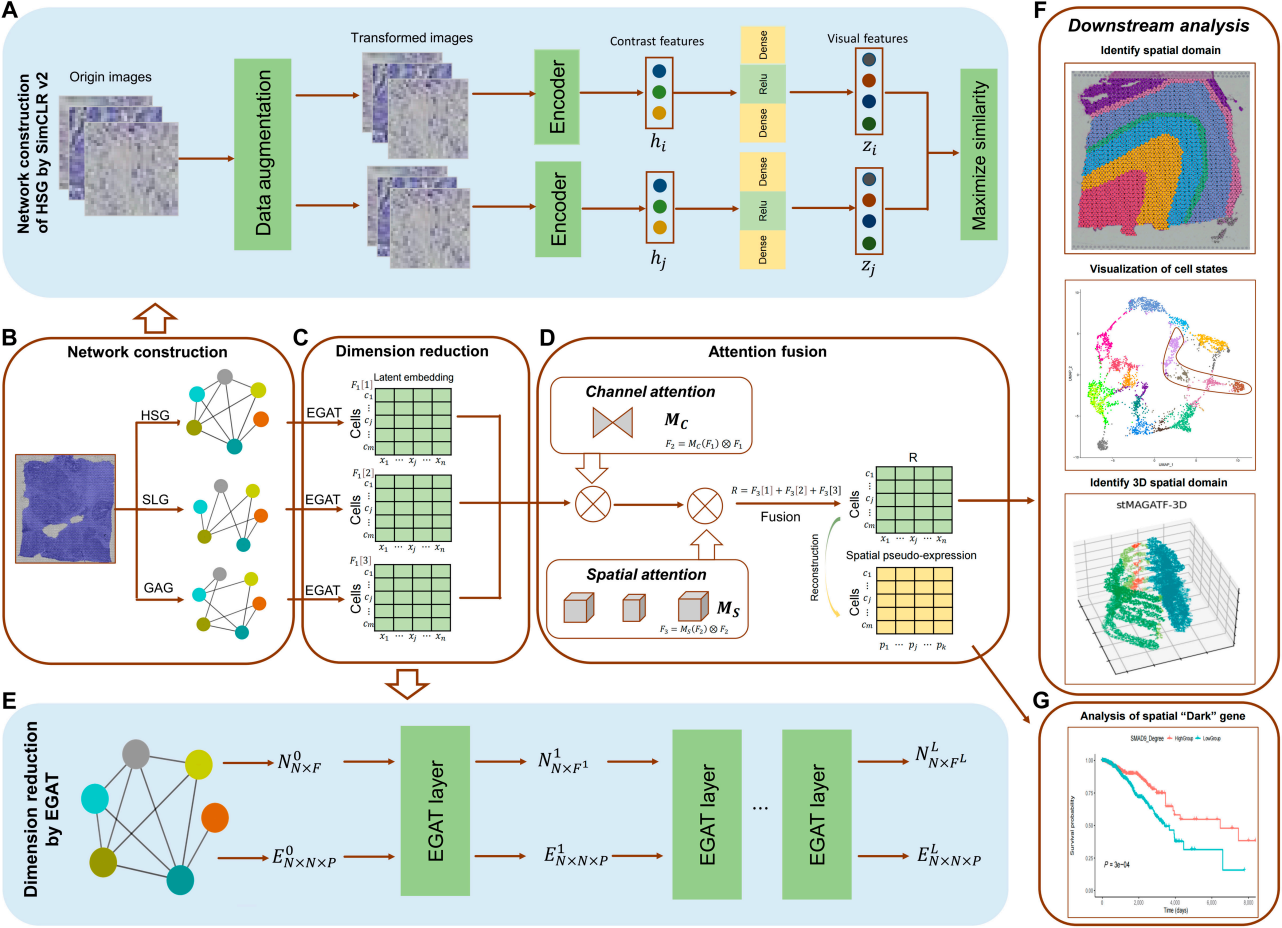
Overview of the stMGATF model. (A) Network construction of HSG by SimCLRv2. Schematic illustration of SimCLRv2 workflow. (B) Network construction. stMGATF constructs HSGs, SLGs, and GAGs using gene expression features learned by the autoencoder framework, histological image visual features extracted by SimCLRv2, physical location, and associations between genes. (C) Dimension reduction. Edge feature enhanced graph neural network (EGAT) is employed to obtain the potential embedding of each view. (D) Attention fusion. Three views are adaptively integrated to obtain low-dimensional representation using GAM and to reconstruct SPE. (E) Dimension reduction by EGAT. Schematic illustration of EGAT architecture. (F) Downstream analysis. stMGATF contributes to detect spatial domain, denoise data, elucidate tissue heterogeneity, reconstruct 3D tissue models and accurate 3D expression domains, and process data of different resolutions. (G) Analysis of SDGs. stMGATF can identify SDGs using reconstructed SPE.

stMGATF first learns visual features of histological images by SimCLRv2 (Fig. 1A) and constructs HSGs, SLGs, and GAGs (Fig. 1B) using gene expression features learned by the autoencoder framework, histological image visual features, physical locations, and associations between genes. EGAT is employed to obtain the potential embedding of each view of the HSGs, SLGs, and GAGs (Fig. 1C). The 3 views are adaptively integrated to obtain low-dimensional representation using GAM, which improves deep neural network performance by reducing information dispersion and amplifying global interaction representations. SPE is obtained from its 15 nearest neighboring points calculated based on the Euclidean distance between any 2 points of the integrated low-dimensional embeddings (Fig. 1D). EGAT can process multidimensional positive-valued edge features for both undirected and directed edges, exploiting a rich source of graph information (Fig. 1E). Exploiting low-dimensional embedding obtained by integration, stMGATF can detect spatial domains and denoise data and contributes to the elucidation of tissue heterogeneity and reconstruction of 3D tissue models and accurate 3D expression domains (Fig. 1F). stMGATF can also process data of different resolutions. Using the reconstructed SPE, stMGATF contributes to the identification of the SDGs, which are nondifferential in the gene expression but sensitive to SPE (Fig. 1G).

### stMGATF improves spatial domain detection performance on the human dorsolateral prefrontal cortex dataset

**Fig. 2.**
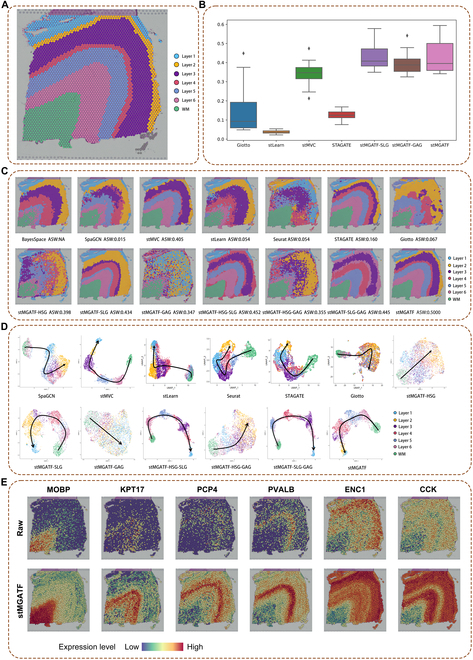
stMGATF improves spatial domain detection performance on the DLPFC dataset. (A) Ground-truth segmentation of cortical layers and WM in DLPFC section 151673. (B) Boxplot of ASW for evaluating the performance of multiview joint features of same cluster compared to Giotto, stLearn, stMVC, STAGATE, stMGATF-SLG, stMGATF-GAG, and stMGATF, on all *n* = 12 samples. In the boxplot, the center line, box limits, and whiskers separately indicate the median, upper and lower quartiles, and 1.5× interquartile range. (C) Cluster assignments generated by BayesSpace, SpaGCN, stMVC, stLearn, Seurat, STAGATE, Giotto, stMGATF-HSG, stMGATF-SLG, stMGATF-GAG, stMGATF-HSG-SLG, stMGATF-HSG-GAG, stMGATF-SLG-GAG, and stMGATF in DLPFC section 151673. (D) Scatter plot of the 2D UMAP extracted from latent features by BayesSpace, SpaGCN, stMVC, stLearn, Seurat, STAGATE, Giotto, stMGATF-HSG, stMGATF-SLG, stMGATF-GAG, stMGATF-HSG-SLG, stMGATF-HSG-GAG, stMGATF-SLG-GAG, and stMGATF in DLPFC section 151673. Note that as end-to-end clustering approaches, BayesSpace cannot be visualized using UMAP. (E) Spatial expression of layer-specific genes: *PCP4*, *PVALB*, *ENC1*, *CCK*, *KRT17*, and *MOBP* for slice 151673 data denoised by stMGATF, where we also provide raw data as a comparison.

We applied the stMGATF model to a dorsolateral prefrontal cortex (DLPFC) 10x Visium dataset to evaluate the performance on spatial domain detection. Maynard et al. [6] sequenced 12 tissue slices and annotated the 6 neuronal layers and white matter (WM) layer in 3 human brains, which allowed us to evaluate the accuracy of spatial domain detection based on the manually annotated tissue layers provided by the original study (Fig. 2A).

Compared with previous spatial clustering methods (BayesSpace, SpaGCN, stMVC, stLearn, Seurat, STAGATE, and Giotto), we found that stMGATF improves the identification of spatial domains. Most SRT methods are unsupervised and use adjusted Rand index (ARI) indices to evaluate the accuracy of spatial domain detection. For a fair comparison, we used the average silhouette width (ASW) index to measure the performance of identifying spatial domains [10]. We plotted the ASW boxplot of DLPFC 12 slices predicted by Giotto, stLearn, stMVC, STAGATE, stMGATF-SLG, stMGATF-GAG, and stMGATF (Fig. 2B) and found spatial domain identification performance evaluated by ASW of stMGATF superior to that of other spatial clustering approaches. Simultaneously, we defined the number of clusters (see Materials and Methods “Clustering” for technical description) and plotted the ARI histograms of various methods applied to the DLPFC section 151673 (Figs. S1 and S2). The evaluation of spatial domain identification performance, based on ARI, reveals that stMGATF outperforms other spatial clustering approaches.

Visualizing the clustering results of the different algorithms on the DLPFC dataset (Fig. 2C), it is apparent that stMGATF is closest to the known layer and achieves the highest clustering accuracy (ASW = 0.5000). stMGATF outperforms other methods at spatial domain detection, as patches belonging to different layers are distinctly separated. We also plotted the clustering results of DLPFC 12 slices predicted by stMGATF-HSG, stMGATF-SLG, stMGATF-GAG, stMGATF-HSG-SLG, stMGATF-HSG-GAG, stMGATF-HSG-GAG, and stMGATF to show the significance of each view (Fig. S4), where stMGATF-HSG is a model where only single-view HSG is constructed and the others are similarly. The stMGATF-HSG-SLG refers to the construction of models that consider 2 specific views, namely, HSG and SLG, as well as other similar views. We found that the integration of the 3 views achieved the best performance on the recognition of the spatial domain.

As a comparison (Fig. 2C), Louvain, a traditional clustering method, had discontinuous clustering boundaries with many outliers. Giotto [4] discriminated spatial domains by incorporating hidden Markov random field models. The boundaries of the Giotto clusters were discontinuous, and there were numerous outliers. stLearn [3] exploits histological information. In the uniform manifold approximation and projection (UMAP) plot generated by stLearn embeddings, spots belonging to different layers are not explicitly separated, mixing spots belonging to layers 3 and 5. BayesSpace clusters with a Bayesian approach use a priori information, but patches belonging to different layers are not distinctly separated [5]. SpaGCN integrates the information on gene expression, spatial location, and histology by adopting a graph convolutional network [7]. The boundaries of the Giotto clusters are discontinuous, and the UMAP mix spots belonging to layers 3 and 4. STAGATE [8], a graph attention autoencoder framework, clusters by learning lower-dimensional features. Generated by STAGATE embeddings, the UMAP mix spots belonging to layers 1 and 3.

Moreover, we performed parameter analyses and 10-fold cross-validation to verify the robustness of stMGATF. We investigated the influence of the hyperparameters μ and λ on 151,673 slices. Figure S3 illustrates ASW results in a 2-dimensional (2D) figure manner. From Fig. S3, we found that the parameters setting μ and λ is critical to the proposed model. Specifically, the highest clustering result occurred when μ = 10 and λ = 200. This phenomenon reflects that the threshold constructing the edges of graph is critical. We use (10,200) as the threshold for defining edges of SLG. In addition, we used 10-fold cross-validation to evaluate the stability of the stMGATF by randomly dividing all the data into 10 roughly equal parts, 9 of which are used for training and the remaining one for testing. The 10-fold cross-validation results showed that the ASW of the stMGATF model was 0.47 and the model was stable (Table S1).

stMGATF can depict spatial trajectories in UMAP due to the integrated spatial information. 2D UMAP scatter plots were drawn from features extracted from SpaGCN, stMVC, stLearn, Seurat, STAGATE, Giotto, stMGATF-HSG, stMGATF-SLG, stMGATF-GAG, stMGATF-HSG-SLG, stMGATF-HSG-GAG, stMGATF-SLG-GAG, and stMGATF (Fig. 2D and Fig. S5). UMAP plots generated from the potential features of stMGATF showed consistent spatial trajectories. It is obvious that the integration of the 3 views achieved the best performance. As a comparison, spots belonging to different layers were not clearly separated in UMAP produced by stLearn and Giotto low-dimensional embedding. Seurat failed to plot a consistent temporal order. STAGATE did not clearly distinguish between layers 3 and 4 and mixed spots from these layers.

stMGATF averages the gene expression data of the 15 nearest cells (determined by the Euclidean distance of the embeddings) to denoise the raw data. Some layer-specific genes were found to be more highly expressed in the SPE compared with the raw gene expression data (Fig. 2E and Fig. S6). For example, *MOBP* is more highly enriched in layer WM, *KRT17* is more highly expressed in layer 6, and *PCP4* is more highly enriched in layer 5 than raw gene expression data [11]. Overall, with these results, the ability of stMGATF to reduce noise and enhance spatial expression patterns is demonstrated.

### Dissecting tumor heterogeneity and identifying cell states using stMGATF

**Fig. 3.**
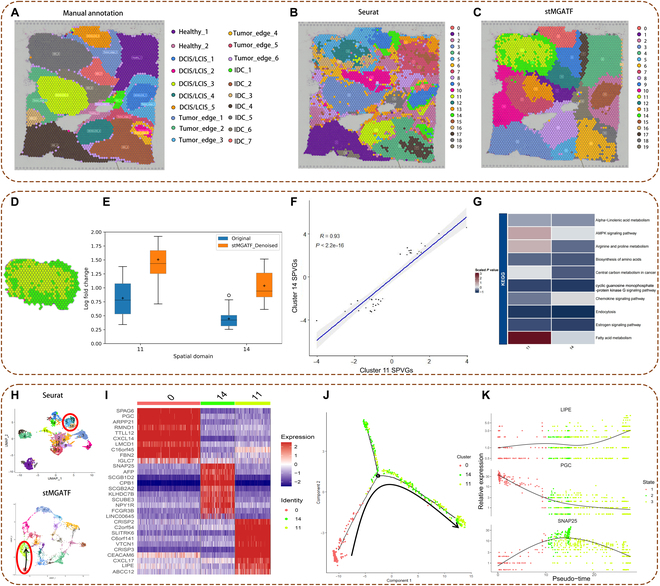
stMGATF can identify and visualize tumor-related cell states from 10x Visium SRT data of human breast cancer. (A) Manual pathological annotation based on hematoxylin and eosin staining of human breast cancer data. (B) Clustering results of Seurat. (C) Clustering results of stMGATF. (D) stMGATF clusters 11 and 14. (E) Log2FC boxplot of SVGs in clusters 11 and 14 before and after stMGATF denoising based on breast cancer data. (F) Scatter plot illustrating the correlation of SPVGs between clusters 11 (tumor region) and 14 (tumor edge) before and after stMGATF denoising. (G) Heatmap showing selected signaling pathways (rows) that were significantly enriched in KEGG analyses for clusters 11 and 14 on stMGATF denoising (columns). (H) UMAP visualization of latent features by Seurat and stMGATF. (I) Heatmap of average gene expression of signature genes from 3 clusters by stMGATF. Rows and columns indicate genes and clusters, respectively. (J) Visualization of clustering and trajectory inference from stMGATF clusters 0, 11, and 14. (K) Pseudo-time-dependent changes in the expression levels of *LIPE*, *PGC*, and *SNAP25*. Each color indicates 1 cluster. AMPK, adenosine monophosphate-activated protein kinase.

To test whether stMGATF can dissect heterogeneities that are distributed at different positions in the tissue, we tested it on the 10x Visium SRT data for human breast cancer. We used hematoxylin and eosin staining for manual pathological labeling [6], and based on the pathological features, we manually segmented the histological images into 20 regions, which were classified into 4 morphological types: ductal carcinoma in healthy tissue (healthy), situ/lobular carcinoma in situ (DCIS/LCIS), invasive ductal carcinoma (IDC), and the area around the tumor with low-characteristic malignancy (tumor edge) (Fig. 3A). We took the marked area as the rough label, randomly selected 70% of the area to weakly supervise the training of stMGATF, and extracted 20-dimensional features from breast cancer. We then predicted cell clusters by Seurat and stMGATF. We found that stMGATF facilitates the detection of more domains enriched for cancer regions. The stMGATF clusters presented a smoother segmentation than Seurat, which appeared fragmented with irregular boundaries. Notably, stMGATF found more subclusters within the tumor regions (Fig. 3B), while Seurat tended to divide the healthy region into subclusters; the methods were set to generate the same number of clusters (Fig. 3C). Specifically, within the seemingly homogeneous tumor region IDC (stMGATF cluster 11), stMGATF separated the tumor edge (stMGATF cluster 14) (Fig. 3D). These stMGATF clusters suggest transcriptionally and spatially distinct compartments within visually homogeneous tumor regions. Additionally, the feature embeddings extracted by stMGATF were better separated between those different domains than those by Seurat, and each domain had its spatially variable genes (SVGs). These results help to further dissect the tumor microenvironment [12].

We perform an analysis to further characterize the transcriptional differences between stMGATF clusters 11 and 14. Figure 3E shows the log2FC boxplot of the top 30 public genes of clusters 11 and 14 before and after stMGATF denoising. These genes are different before and after denoising with stMGATF. We observe that stMGATF-denoised log2FC performs better than the original data. Higher values of log2FC indicate genes easier to detect. stMGATF improves the degree of fold change between genes, and some new differential genes can be found, which would otherwise be missed, by evaluating whether the average expression levels of the signature genes between 2 domains are different or not correlated. There was a strong correlation between clusters 11 and 14 before and after stMGATF denoising, indicating that the status of adjacent tumor tissue was partially reflected in the invasive front (*R* = 0.93, *P* < 0.001; Fig. 3F). Overall, cluster 14 represented a region with an immune-suppressed protumor microenvironment and high potential for cancer metastasis. To characterize the potential functions of clusters 11 and 14, we performed Gene Ontology (GO) and Kyoto Encyclopedia of Genes and Genomes (KEGG) enrichment analyses for spatial pseudo-variable genes (SPVGs) (Fig. S7 and Table S2). We observed a distinct pattern of pathway enrichment for these clusters, suggesting their various functions. Particularly, endocytosis was highly enriched (Fig. 3G). Endocytosis of *GPR54* and *EGFR* is modulated by *EGF* or *KP-10* stimulation in breast cancer cells [13]. A new dysregulation point that plays a role in tumor metastasis can be identified through altered signaling of endocytosis. Furthermore, multiple endocytic proteins are abnormally regulated in tumors and modulate tumor metastasis, especially migration and invasion, suggesting that there may be cell migration between clusters 11 and 14.

The process of onset and development of tumor tissue heterogeneity is dynamic, and trajectory inference reconstructs this process by finding pathways that minimize transcriptome differences between neighboring cells [14]. The low-dimensional features extracted by stMGATF are used to visualize the data with UMAP. We observed that the 3 clusters detected by stMGATF are very different from Seurat, predicting the possible trajectories between the 3 domains outlined with red, i.e., cluster 0 → cluster 14 → cluster 11, while there is no direct trajectory in the 3 clusters of Seurat (Fig. 3H).

We validated our predictions as follows. The feature embeddings extracted by stMGATF are better separated between different domains, each with specific signature gene SVGs (Fig. 3I). Three clusters have important functions in the metastatic dissemination of breast cancer. We performed pseudo-time trajectory analysis using Monocle2 [15] between 3 clusters based on breast cancer data, which revealed the pseudo-time status for clusters 0, 11, and 14 (Fig. 3J and Fig. S8). Cluster 0 cells show overexpression of *PGC* that could be correlated with the regulating metabolic reprogramming in cancer cells [16]. *PGC* promotes in vitro migration and invasion of breast cancer cells and in vivo lung metastasis [17]. Cluster 14 cells with overexpression of *SNAP25* mediate cell invasion and apoptotic process [18]. Overexpression of *LIPG* in cluster 11 cells is significantly associated with tumor formation and metastasis in human breast cancer [19] (Fig. 3K). With the estimated pseudo-time, the expression of *PGC* for cluster 0 decreases, LIPE for domain 11 increases, and *SNAP25* for domain 14 is highly expressed at the middle state, supporting our trajectory inference between clusters 0, 11, and 14. These results indicate possible cell development from tumor edge cells to malignant tumor cells. At the same time, we analyzed stMGATF on IDC samples from another breast cancer data and identified a possible trajectory of cluster 1→cluster 13→cluster 8 (Fig. S9), which is consistent with the findings in previous studies [9]. Taken together, stMGATF can identify cell states.

### stMGATF improves 3D spatial domain detection performance on the Slide-seq datasets using 3D location information

**Fig. 4.**
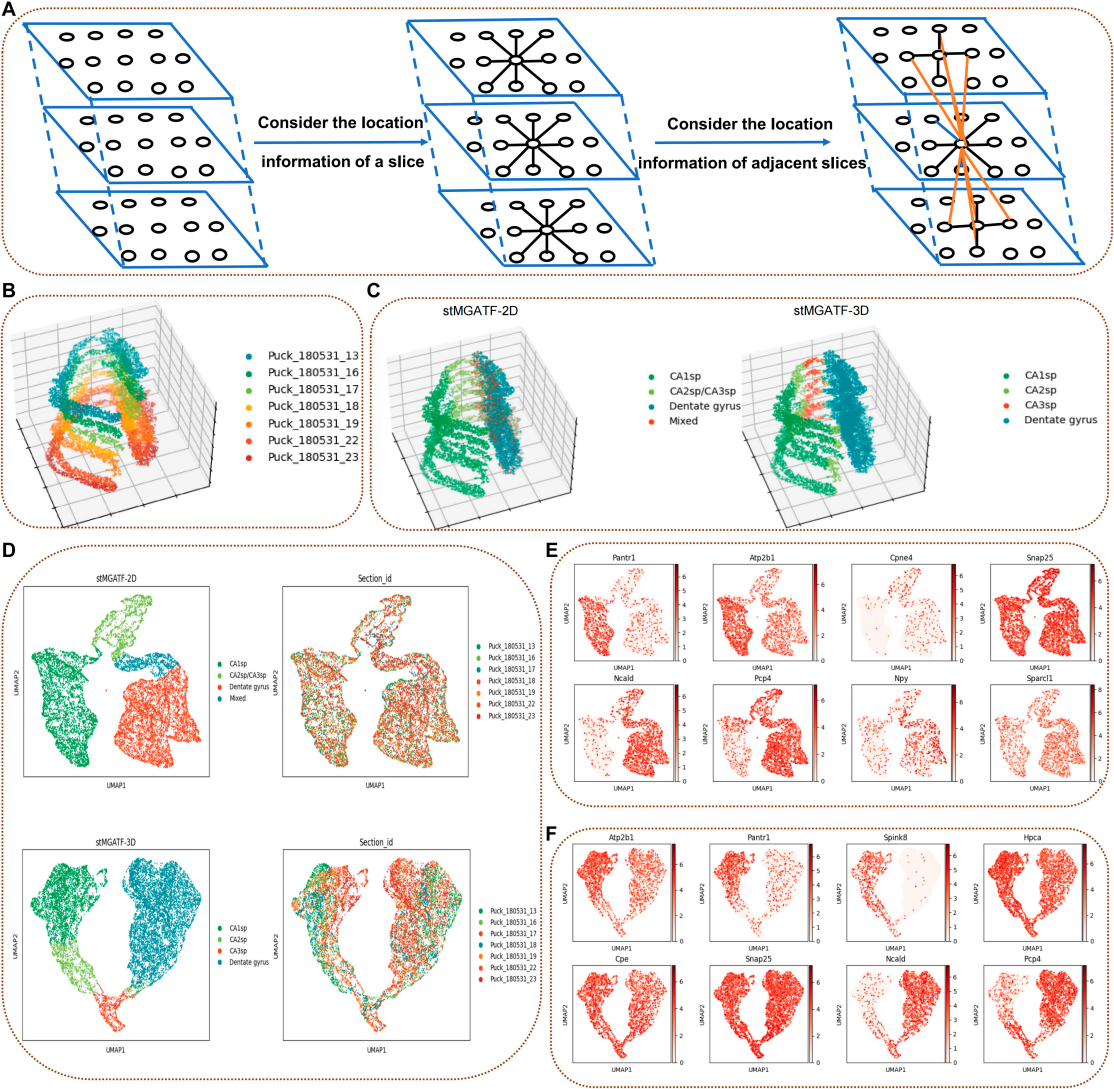
stMGATF improves 3D spatial domain detection performance by constructing an stMGATF-3D network, alleviating the batch effect between neighbor slices. (A) Multiple contiguous slice information is exploited to construct stMGATF-3D, which alleviates the batch effect between neighbor slices. (B) Visualization of cord-like structure extracted from 3D hippocampal volume of 7 consecutive slices stacked as described by Slide-seq. (C) Visualization of spatial domain detection by stMGATF-2D and stMGATF-3D. (D) UAMP plot generated by stMGATF-2D and stMGATF-3D. (E) UMAP visualization of marker genes *Pantr1*, *Atp2b1*, *Cpne4*, *Snap25*, *Ncald*, *Pcp4*, *Npy*, and *Sparcl1*. (F) UMAP visualization of marker genes *Atp2b1*, *Pantr1*, *Spink8*, *Hpca*, *Cpe*, *Snap25*, *Ncald*, and *Pcp4*.

stMGATF-3D achieves profiling gene expression patterns in a 3D context through consideration of multiple contiguous slice information, using multidimensional edge information to construct a 3D spatial graph, which alleviates the batch effect of adjacent and reconstructed 3D tissue models and extracted 3D expression domains (Fig. 4A). We applied the stMGATF model to pseudo-3D SRT data [8], which was constructed by aligning the spots of the cord-like structure in 7 hippocampus sections profiled by Slide-seq (Fig. 4B). Rodriques et al. [20] sequenced pucks capturing 66 sagittal tissue sections from a single dorsal mouse hippocampus, which allows us to exploit the gene expression and location information to identify spatial domains. We found that stMGATF suffers from identifying CA2sp domains when only 2D tissue slice information was used, due to batch effects between slices, while stMGATF-3D clearly portrays known tissue structures after the addition of adjacent edges between adjacent slices (Fig. 4C). By using the location information of spatially adjacent slices, UMAP based on clustering results and slice IDs were drawn using the low-dimensional features extracted by stMAGTF. UMAP based on stMGATF-3D clustering results preferred to be clustered by stMGATF-2D (Fig. 4D). stMGATF-3D effectively detects layer-specific genes and contributes to identifying layer-specific genes of each layer based on the result predicted by stMGATF (Fig. 4E and F and Figs. S10 and S11).

### stMGATF reveals the layer-specific marker genes in mouse visual cortex STARmap data

**Fig. 5.**
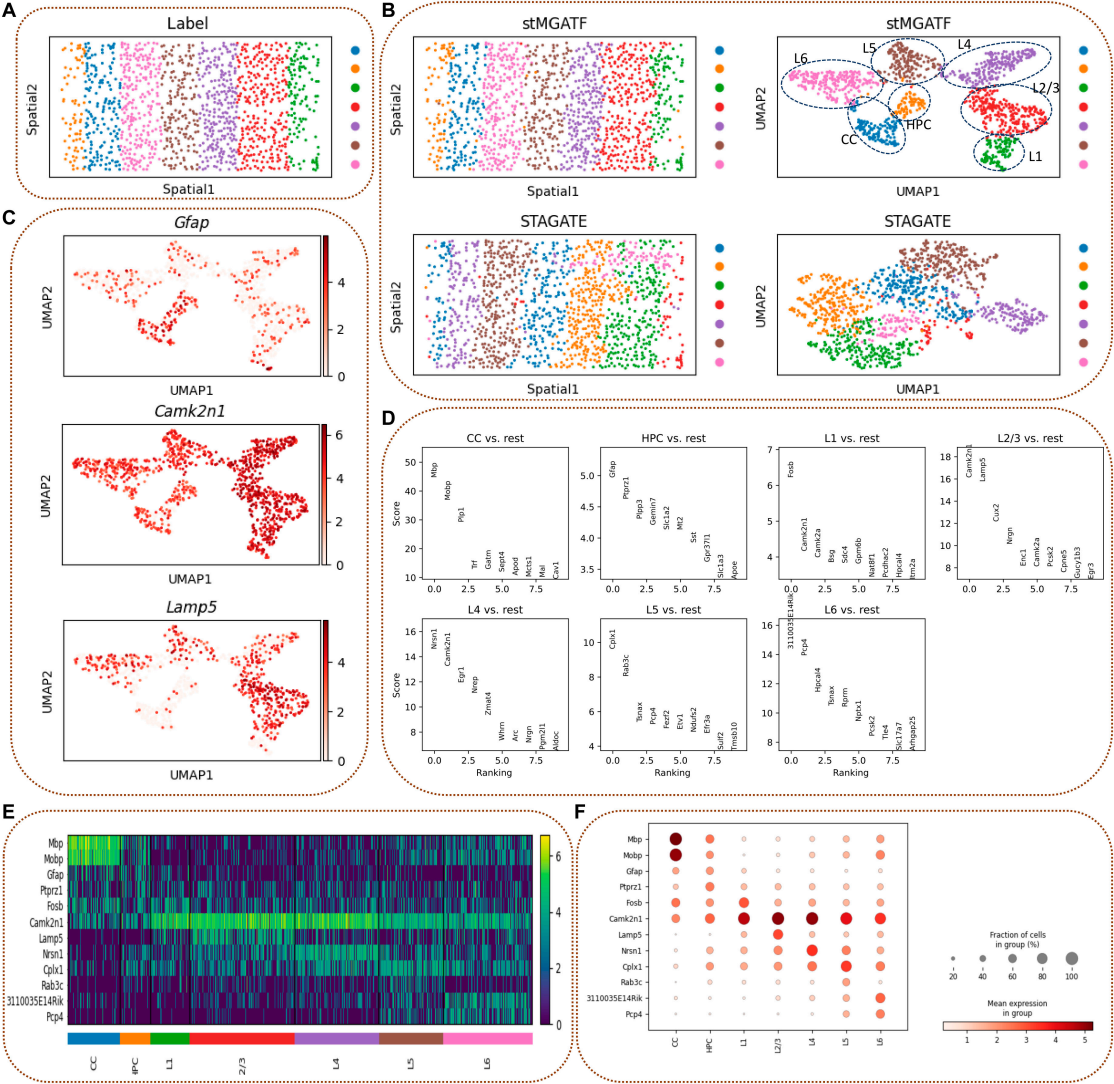
stMGATF improves the performance of layer-specific inhibitory neuron detection in mouse primary visual cortex. (A) Layer structure and cell type distribution of mouse primary visual cortex (V1) dataset. (B) Spatial domains predicted by stMGATF and STAGATE; UMAP visualization of latent embedding extacted by stMGATF and STAGATE. (C) UMAP visualization of marker genes *Gfap*, *Camk2n1*, and *Lamp5*. (D) Visualization of top 10 marker genes in each layer. (E) Heatmap of top 2 marker genes in each layer. (F) Dot map of top 2 marker genes in each layer.

We applied stMGATF to the mouse visual cortex dataset profiled by STARmap, which has a single-cell resolution. Wang et al. [21] generated the dataset from mouse visual cortex, which spans from the hippocampus to the corpus callosum, including the 6 neocortical layers (Fig. 5A). stMGATF reveals spatial expression patterns of SPVGs detected by stMGATF (Fig. S12) and, more importantly, improves the performance of spatial domain detection. As a comparison, we extracted the latent features by STAGATE to identify the spatial domain. The ARI value for stMGATF is 0.8075, and for STAGATE it is 0.5441; the ASW value for stMGATF is 0.4464, and for STAGATE it is 0.1059. stMGATF can depict spatial trajectories in UMAP due to the integrated spatial information. Two-dimensional UMAP scatter plots were drawn from features extracted from STAGATE and stMGATF. UMAP plots generated from latent features of stMGATF showed consistent spatial trajectories. As a comparison, spots belonging to different layers were not clearly separated in UMAP produced by STAGATE low-dimensional embedding (Fig. 5B). Importantly, stMGATF detects layer-specific genes of each layer based on the predicted result. *Gfap* is highly expressed in layer CC, is a marker protein of astrocytes, and is uniquely and abundantly expressed in astrocytes. Central nervous system injury can result in an astrocytic glial response, which leads to enhanced expression of *Gfap*. Therefore, *Gfap* acts as a biochemical marker of the extent of central nervous system injury and prognosis [22]. Moreover, *Gfap* expression occurs abnormally in gliomas and thus can be a specific marker and reflect the malignancy of gliomas [23]. *Camk2n1* is enriched in layers 4 and 2/3. Calcium ion/calmodulin-dependent protein kinase II inhibitor (*Camk2n1*) is an endogenous calmodulin-dependent protein kinase II inhibitor. *Camk2n1* appears to be a prognostic marker in ovarian cancer [24] and is tumor-suppressive in prostate cancer [25] and glioma [26]. Reduced expression of *Camk2n1* correlates with the progression of DCIS to invasive breast cancer [27]. *Lamp5* is highly expressed in layer 1. *Lamp5* has a crucial role in sensory-motor processing in the brainstem and spinal cord [28] and is localized only in inhibitory synaptic terminals [29]. *Lamp5* is highly expressed in several brainstem nuclei involved in auditory processing, including the cochlear nucleus, superior oligomeric complex, lateral meningeal nucleus, and spinal cord gray matter (Fig. 5C). *Cplx1* is enriched in layer 5 and is most abundantly expressed in dendrites [30] (Fig. 5D). We plotted a heatmap and dot map to visualize the expression of layer-specific genes (Fig. 5E and F).

### SDGs revealed by stMGATF

**Fig. 6.**
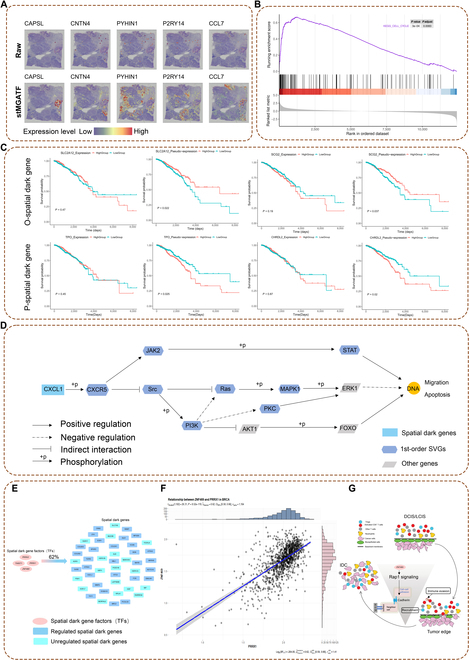
stMGATF can identify SDGs. Validation and analysis of SDGs are also shown. (A) Spatial expression of SDGs for IDC region denoised by stMGATF; we provide raw data as a comparison. (B) GSEA using SDGs. (C) Comparison of survival curves of O-SDGs and P-SDGs by stMGATF in RNA-seq data of breast cancer from TCGA database. (D) Regulation of related SDGs in the chemokine signaling pathway. (E) Top 4 upstream TFs (*PRRX2*, *TWIST1*, *PRRX1*, and *ZNF469*) could regulate 62% of SDGs that were identified by clusters 11 and 14 based on breast cancer data. (F) Correlation scatter plot of *ZNF469* and *PRRX1* expression in BRCA with lateral histogram. (G) Schematic of the immune escape mechanism, where *ZNF469* gains the ability to recruit immunosuppressive Treg cells through a positive feedback loop of Rap1 signaling, thereby promoting the formation of a metastatic microenvironment conducive to metastasis.

Traditional methods mainly focus on SVGs, which are only differential in the gene expression level. Considering the associations between genes, stMGATF reconstructs the SPE. It can find the SVGs capable of identifying SPVGs, which are sensitive and differential in SPE. We focus on SDGs from SPVGs, which are differential in SPE but undifferential in gene expression between different clusters (i.e., not belonging to SVGs). More importantly, these SDGs were found to be closely associated with disease progression. For example, SDG *CCL7* (Fig. 6A) showed an obvious difference between cluster 0 and other clusters based on SPE, while its raw spatial expression is completely messy and shows no obvious difference, indicating the ability of stMGATF to reveal biological variation among tumor areas.

To further validate the relationship between SDGs and breast cancer, we performed gene set enrichment analysis (GSEA) with 169 SDG set permutations. Remarkably, GSEA (Fig. 6B) revealed a significant positive correlation between SDGs and KEGG_CELL_CYCLE pathways, indicating a dominant place in the anti-inflammatory role of transcriptional programs related to cell cycle regulation [31].

In addition, the prognostic significance of the subgroup of the SDG *SLC2A12* based on the SPE and gene expression are 0.022 and 0.47, respectively, which means that the different subgroups obtained based on the SPE can stratify breast cancer patients into subgroups with different survival times well from the viewpoint of statistics, while the gene expression failed. This indicates the validity of stMGATF in breast cancer diagnosis. For other SDGs, we also obtained a similar result. The SDGs can be further divided into optimistic SDGs (O-SDGs) and pessimistic SDGs (P-SDGs). Figure 6C shows some O-SDGs and P-SDGs for independent breast cancer patients from The Cancer Genome Atlas (TCGA) database. We discovered that samples with higher SPE of O-SDGs (i.e., *SLC2A12* and *SCG2*, *P* < 0.05) survive significantly longer than those with low values, while samples with higher SPE of P-SDGs (i.e., *TPO* and *CHRDL2*, *P* < 0.05) survive for significantly less time than those with low values (Fig. 6C). We also performed a functional analysis of SDGs (Fig. S13), which are enriched in pathways that are closely associated with breast cancer. For instance, in breast cancer cells, transduction through the chemokine signaling pathway mediates actin polymerization and pseudopod formation, which subsequently induces chemotactic and invasive responses [32]. It is seen that the SDG *CCL7* is an upstream regulatory gene that promotes early breast cancer survival and invasion through a fibroblast-dependent mechanism in the chemokine signaling pathway (Fig. 6D).

Because TFs are key players that drive shifts in cell state properties [33–35], we used ChEA3 to predict the TFs of SDGs for clusters 11 and 14 in breast cancer data [36]. The hub TFs (*PRRX2*, *TWIST1*, *PRRX1*, and *ZNF469*) could regulate 62% of SDGs (Fig. 6E and Fig. S15). Among these 4 upstream TFs, *PRRX2*, *TWIST1*, and *PRRX1* were reported to induce mesenchymal–epithelial cell transformation and were associated with breast cancer metastasis [37–39]. These TFs may play a key regulatory role in cell proliferation, invasion, and migration. SDGs predict TFs associated with extracellular matrix structures, integrin binding, and cell adhesion. TFs are involved in multiple signaling pathways and are associated with tumorigenesis (Table S3). The cell adhesion molecule pathway has an important role in cell metastasis and can be divided into calmodulin, integrins, and selectins. They maintain intercellular connections in epithelial tissues, participate in the epithelial–mesenchymal transition process, prevent cell detachment and invasion into surrounding tissues, promote cell differentiation, and inhibit proliferation [40]. In the level of SPE of *PRRX2*, the survival rate of patients is significantly different (*P* < 0.05; log-rank test) (Fig. S16), demonstrating that stMGATF can identify hub upstream TFs closely associated with good prognosis in breast cancer patients through SDGs.

While the role of *ZNF469* in breast cancer differentiation has not been previously investigated, we found that it promotes metastasis from tumor edge cells to malignant tumor cells via Rap1. *ZNF469* is a DNA-binding protein consisting of a zinc finger structural domain that regulates the expression of many genes involved in various biological processes, including cell adhesion, migration, and differentiation [41]. We show a correlation between *ZNF469* and breast cancer metastasis-related TF expression from RNA sequencing (RNA-seq) data of breast cancer in TCGA database (Fig. 6F and Fig. S17). *PROX1* interacts with *hnRNPK* to inhibit the ubiquitination of *hnRNPK*, which in turn activates the WNT pathway to promote invasive metastasis of breast cancer [42]. As shown in Fig. 6F and Fig. S17, Z*NF469* expression was positively correlated with *PRRX1* expression levels in breast invasive carcinoma (BRCA) (${\hat{r}}_{\text{Pearson}}$ = 0.62, *P* < 0.001), which suggests that *ZNF469* may be associated with tumor metastasis, affecting breast cancer progression, and may provide useful information for early clinical diagnosis, new therapies, and targeted treatments. The Rap1 signaling pathway regulates integrins and cadherins, which play important roles in cell adhesion to the extracellular matrix and cell–cell adhesion [43]. Therefore, the tumor edge is a region of the tumor microenvironment and has high cancer metastatic potential. Immune escape is an important mechanism in tumor progression, where tumor cells are able to evade the immune system in various ways. We could find *ZNF469* as one of the zinc finger protein family members through SDGs. The zinc finger protein family gains the ability to recruit immunosuppressive regulatory T (Treg) cells through a positive feedback loop of Rap1 signaling, which can promote the formation of a microenvironment conducive to tumor metastasis [44]. Treg cells are an immunosuppressive cell type that can inhibit the activation and attack of other immune cells, thus helping tumor cells to evade immune surveillance. This sheds some light on *ZNF469* as a possible promotion of metastatic microenvironment formation via the Rap1 signaling pathway (Fig. 6G). *ZNF469* uses multiple immune evasion strategies that may include immunosuppression of chemokine production. Notably, chemokines ranked highest in the differentially upregulated transcriptome associated with immune evasion [45], suggesting a key role in *ZNF469* immune escape. *ZNF469* has the potential to provide a diagnostic and drug target for the prevention and treatment of breast cancer.

## Discussion

Accurate identification of spatial domains and further extraction of spatially expressed genes are essential for understanding tissue organization and biological functions. We developed a spatial domain identification method, stMGATF, which integrates gene expression, histological images, spatial location, and gene association.

stMGATF exploits EGAT to learn a cell embedding representation based on graphs extracted from SRT data and simultaneously integrates 3-view graphs for robust cell embedding representation through a global attention fusion mechanism, which can be used to identify spatial domains, denoise original data, and conduct further downstream analyses. We tested the performance of stMGATF on diverse SRT data generated with different platforms and spatial resolutions and found that the representation learned by stMGATF is robust to accurately revealed the hierarchical structure and organizational heterogeneity of SRT data. We illustrated the ability of stMGATF to alleviate batch effects for consecutive sections. Experimental results suggest that stMGATF is a promising algorithm to improve understanding of cell identity, interaction, and spatial organization from SRT data.

Unlike the assumption made in most approaches, which only consider spatial and gene expression-level information [46,47], stMGATF considers histology images and associations between cells across the tissue sample. Some methods use histological images but have limitations. SpaGCN [7] uses histological image pixels as features by directly calculating the mean color values from the RGB channels. However, the pixel values are easily affected by noise and cannot provide semantic features for cell analysis. Our method has advantages including modularization, interactive visualization, reproducibility, robustness, and flexibility. stMGATF is specifically targeted for SRT data analysis. In real data analysis, relevant views can be selectively constructed for cell embedding representation. stMGATF can facilitate the identification of tissue organization and the discovery of corresponding gene markers. With the advance of spatial multiomics technology [48], such integration of multiview graph attention fusion mechanisms may greatly enhance the mechanistic understanding of cell state variation in development and diseases. Cell embedding representations of 3 views can be selectively constructed for application to the construction of views of different data in practical data applications. SDGs play an important role in tumor metastasis, and the predicted TFs are closely related to the tumor microenvironment and immune escape, suggesting that SDGs may be involved in various functional and signaling pathways affecting breast cancer progression, and may provide useful information for early clinical diagnosis, new therapies, and targeted treatments [49].

Nonetheless, our work has limitations: (a) How to accurately select the number of clusters remains a limitation when applying stMGATF to new datasets without prior knowledge, which is a problem for most clustering methods. (b) Breast cancer is a complex disease, and more experimental and clinical studies are still needed to validate and deeply explore the regulatory mechanisms of SDGs in breast cancer.

stMGATF is a promising new approach that jointly learns the robust cell embedding representation by integrating gene expression, image morphology, spatial information, and gene association. Soon, we expect to improve its applicability through its usage on SRT datasets of different resolutions generated by new technologies.

## Materials and Methods

### Using an autoencoder framework to extract feature matrix

For gene expression data, we learn its *K*-dimensional feature *z* by an autoencoder (𝐸) [50] and convert *z* into the parameters *D_u_* and *D_θ_* of the negative binomial distribution by the corresponding decoder (𝐷) to obtain the feature matrix *X*^0^:

$$\begin{array}{l} p(x|z)=\mathrm{NB}(x;u_{x},\theta_{x})=\mathrm{NB}(x,l_{x};D_{u}\left(z\right),D_{\theta }\left(z\right)),\\ \mathrm{NB}(x;u_{x},\theta_{x})=\frac{\Gamma \left(x+\theta_{x}\right)}{\Gamma \left(\theta_{x}\right)\Gamma \left(x+1\right)}{\left(\frac{u_{x}}{u_{x}+\theta_{x}}\right)}^{x}{\left(\frac{\theta_{x}}{\theta_{x}+u_{x}}\right)}^{\theta_{x}} \end{array}$$

where *u_x_* and *θ_x_* are the mean and dispersion, respectively, of the negative binomial distribution of each gene in the SRT data, and *D_u_* and *D_θ_* can be inferred using the softmax activation function in the last layer of the neural network. The 1-dimensional constant *l_x_* is calculated as a cell-specific normalization factor after the sum of the counts of the selected genes in each cell. The training objective of the model is to maximize the marginal likelihood of the observed gene expression data with the following loss function:

$$\text{log}\;p(x|z)=E_{z∼p(z|x,E)}(\text{log}\;p(x|z);D_{u},D_{\theta })$$

The neural network has 2 fully connected layers with 1,000 nodes, using dropout regularization and batch normalization, with a ReLU activation function between the 2 hidden layers, and an Adam optimizer with 1𝑒−6 weight decay and an 8𝑒−05 learning rate to minimize the loss function. To reduce computational stress, we used 2,000 highly variable genes to capture the internal structure of the gene expression data, so the autoencoder frame was fixed to [2,000, 1,000, 50, 1,000, 2,000].

### Learning visual features by SimCLRv2

SimCLRv2 learns a general representation of images in an unlabeled dataset and fine-tunes it using a limited number of labeled images to achieve good performance at a classification task [51]. It learns a general representation by adopting contrastive learning, which maximizes consistency between transformed views of the same image and minimizes consistency between those of different images. This comparison objective updates the parameters of the neural network so that the representations of corresponding views are mutually attracted, while noncorresponding views are mutually excluded. The model is described as follows:

1. We used unlabeled data to pretrain the encoder in a task-agnostic way to obtain a comparison of general representations.

First, we performed data enhancement for each image, obtaining 2 enhancement results for each image, with the data processing methods of random cropping followed by resizing back to the original size, random color distortions, and random Gaussian deblur.

Taking 2 pictures as examples, we input pictures *x_i_* and *x_j_* into 2 encoders that share parameters and obtain representations *h_i_* and *h_j_* and continue to obtain representations *z_i_* and *z_j_* through 2 projection heads that share parameters,

$$z_{i}=g\left(h_{i}\right)=W^{\left(2\right)}\sigma \left(W^{\left(1\right)}h_{i}\right).$$

Then, we maximize *z_i_* and *z_j_* as obtained from the same image. Using cosine similarity, the similarity between augmented images *x_i_* and *x_j_* is transformed into the similarity between projected representations *z_i_* and *z_j_*,

$$s_{i,j}=\frac{z_{i}^{T}\;z_{j}}{\;\tau ∥z_{i}∥∥z_{j}∥},$$

where τ is an adjustable temperature parameter that can scale the input and expand the range of cosine similarity [−1, 1]. SimCLR uses a contrastive learning loss function, normalized temperature-scaled cross-entropy loss (NT-Xent). The goal is to maximize the result of softmax, so the loss function is its negative log,

$${l(i,j)}^{\mathrm{NT}-\text{Xent}}=-\log\frac{\exp\left(s_{i,j}\right)}{\sum_{K=1}^{2N}1\left[k!=i\right]\exp\left(s_{i,j}\right)}.$$

Finally, we calculate the average of the sum of losses for all pairs in each batch,

$$L=\frac{1}{2N}\sum_{k=1}^{N}\left[l\left(2k-1,2k\right)+l\left(2k,2k-1\right)\right],$$

and *L* can be optimized.

2. We fine-tune the encoder in a task-specific way using labeled data. In SimCLRv2, after pretraining, only half of the projection head is discarded, and the other half is kept for fine-tuning.

3. We use unlabeled data for knowledge distillation, which can result in a smaller encoder. We use the fine-tuned network as a teacher to distill a smaller student network. The loss function is$L^{\text{distill}}=-\sum_{x_{i}\in D}\;\left[\sum_{y}P^{T}\left(y|x_{i};\tau \right)\log P^{S}\left(y|x_{i};\tau \right)\right].$ *f ^task^*(*x_i_*)[*y*] measures the similarity between the network output and *y* for input image *x_i_*. Hence, *P*(*y*│*x_i_*) is the probability that the network output is similar to *y*, given input image *x_i_*. *P^T^*(*y*│*x_i_*) is the probability of the teacher, while *P^T^*(*y*│*x_i_*) is the probability of the student, and we hope that they are as close as possible.

Compared to SimCLRv1 [52], we use a deeper and wider network model. We fine-tune it with a labeled dataset after pretraining with an unlabeled dataset and distill the model into a smaller network, as the large model is no longer needed once the downstream task is determined, and model accuracy and portability can be improved. We keep the deeper projection head, which can learn better representations, for better fine-tuning of downstream tasks.

### Processing morphological information to construct HSG

To eliminate noise in histological staining and extract morphological information from histological image data, we efficiently learn the visual features ***v***_***i***_ of each point using the SimCLRv2 model [51], maximizing the consistency between different enhanced views of the same point image through the contrast loss in the potential space. Using gene expression features as node features, the visual features extracted by SimCLRv2 are processed to generate 3D edge features to construct the HSG, and the edge features are defined as

$$\begin{array}{c} e_{\mathrm{ij}}^{1}=\frac{v_{i}\cdot v_{j}}{\sqrt{{\left|v_{i}\right|}^{2}+{\left|v_{j}\right|}^{2}}},\\ {\hat{e}}_{\mathrm{ij}}^{2}=\sqrt{{\left(v_{i}-v_{j}\right)}^{2}},\\ e_{\mathrm{ij}}^{2}=e^{-{\hat{e}}_{\mathrm{ij}}^{2}},\\ e_{\mathrm{ij}}^{3}=\frac{\mathrm{cov}\left(v_{i}\cdot v_{j}\right)}{\sqrt{\mathrm{Var}\left[v_{i}\right]\mathrm{Var}\left[v_{j}\right]}}, \end{array}$$

where || denotes the norm of a vector, *Cov* is the covariance between 2 vectors, *Var* is the variance of a vector, and $e_{\mathrm{ij}}^{1},e_{\mathrm{ij}}^{2},e_{\mathrm{ij}}^{3}$ represent the edge features.

### Processing spatial location information to construct SLG

The location information provided by SRT data is a bridge that connects gene expression messages with tissue image messages, complementing the gene expression information and tissue image information. Exploiting spatial information helps to effectively use SRT information. We use gene expression features as node features and process the physical location (*s_ix_*, *s_iy_*) to form 3D edge features and construct a SLG, where edge features are defined as

$$\begin{array}{c} e_{\mathrm{ij}}^{1}=\frac{\left(s_{\mathrm{ix}},s_{\mathrm{iy}}\right)\cdot \left(s_{\mathrm{jx}},s_{\mathrm{jy}}\right)}{\sqrt{{\left(s_{\mathrm{ix}}-s_{\mathrm{jx}}\right)}^{2}+{\left(s_{iy}-s_{jy}\right)}^{2}}},\\ {\hat{e}}_{\mathrm{ij}}^{2}=\sqrt{{\left(s_{\mathrm{ix}}-s_{\mathrm{jx}}\right)}^{2}+{\left(s_{iy}-s_{jy}\right)}^{2}},\\ e_{\mathrm{ij}}^{2}=\left\{\begin{array}{c} 1,{\hat{e}}_{\mathrm{ij}}^{2}\leq \lambda \\ 0,{\hat{e}}_{\mathrm{ij}}^{2}>\lambda , \end{array}\right.\\ {\hat{e}}_{\mathrm{ij}}^{3}=|s_{\mathrm{iy}}-s_{\mathrm{jy}}|,\\ e_{\mathrm{ij}}^{3}=\left\{\begin{array}{c} 1,{\hat{e}}_{\mathrm{ij}}^{3}>\mu \\ 0,{\hat{e}}_{\mathrm{ij}}^{3}\leq \mu , \end{array}\right. \end{array}$$

where || indicates the absolute value operation, μ and λ are hyperparameters, and $e_{\mathrm{ij}}^{1},e_{\mathrm{ij}}^{2},\text{and}\;e_{\mathrm{ij}}^{3}$ represent the 3D features of edges.

### Constructing GAG by exploiting gene association matrix

It has been found that the use of gene association data can substantially improve the performance of single-cell clustering [53]. We employ the cellular association matrix of the SRT data to construct a direct association network between genes for each cell. We determine the conditional independence of genes *x* and *y* given the conditional gene *z* in cell *k* using the statistical index

$$\rho_{\mathrm{xy}|z}^{\left(k\right)}=\frac{n_{\mathrm{xyz}}^{\left(k\right)}}{n_{z}^{\left(k\right)}}-\frac{n_{\mathrm{xz}}^{\left(k\right)}}{n_{z}^{\left(k\right)}}∙\frac{n_{\mathrm{yz}}^{\left(k\right)}}{n_{z}^{\left(k\right)}},$$

which ranges from −1 to 1, where $n_{z}^{\left(k\right)},n_{\mathrm{xz}}^{\left(k\right)},\;n_{\mathrm{yz}}^{\left(k\right)},\text{and}\;n_{\mathrm{xyz}}^{\left(k\right)}$ are the respective numbers of cells in the neighborhoods of *z_k_*, (*x*_*k*,_*z_k_*), (*y*_*k*,_*z_k_*), and (*x*_*k*,_*y*_*k*,_*z_k_*). Genes *x* and *y* in cell *k* are associated, and an edge is present between them in the case that $\rho_{\mathrm{xy}}^{\left(k\right)}$ is greater than the significance level. Conversely, genes *x* and *y* in cell *k* are independent, with no edges between them. To reduce the computational costs, we identify direct associations between pairs of genes in a cell with a small number of conditional genes, which may be key regulatory genes in biological processes, such as TFs and kinases. From the view of the network, the conditional genes are usually the hub genes in the gene–gene network. The default parameters used in constructing the network are *alpha* = 0.5, *kk* = 1, *boxsize* = 1.5, and *weighted* = 1. These can be adjusted according to the situation.

The conditional cell-specific network obtained for cell *k* under conditional gene *z* is denoted as $c_{\mathrm{ij}}^{\left(k\right)}$. We can reduce the dimensionality while integrating the network properties. The conditional network degree of the *i*th gene in the *k*th cell is

$$v_{\mathrm{ik}}=\sum_{j=1}^{m}\;c_{\mathrm{ij}}^{\left(k\right)}.$$

We can obtain a conditional degree matrix containing *m* × *n* elements. Next, we extracted low-dimensional potential embeddings from the conditional degree matrix. To achieve this, we processing the conditional degree matrix *V* = {*v_ik_*|*i* = 1, 2, …, *num*(*gene*), *j* = 1, 2, …, *num*(*cell*)} with an autoencoder. We construct the GAG by processing the gene expression features *w_i_* to form 3D edge features and using the conditional degree matrix features as node features, defined as

$$\begin{array}{c} e_{\mathrm{ij}}^{1}=\frac{w_{i}\cdot w_{j}}{\sqrt{{\left|w_{i}\right|}^{2}+{\left|w_{j}\right|}^{2}}},\\ {\hat{e}}_{\mathrm{ij}}^{2}=\sqrt{{\left(w_{i}-w_{j}\right)}^{2}},\\ e_{\mathrm{ij}}^{2}=e^{-{\hat{e}}_{\mathrm{ij}}^{2}},\\ e_{\mathrm{ij}}^{3}=\frac{\mathrm{Cov}\left(w_{i}\cdot w_{j}\right)}{\sqrt{\mathrm{Var}\left[w_{i}\right]\mathrm{Var}\left[w_{j}\right]}} \end{array}$$

where || indicates the modulus of a vector, and $e_{\mathrm{ij}}^{1},e_{\mathrm{ij}}^{2},\text{and}\;e_{\mathrm{ij}}^{3}$ are the 3D features of edges.

### Learning low-dimensional representations of each view by EGAT

We use EGAT to learn low-dimensional representations in stMGATF, which is capable of exploiting multidimensional positive real-valued edge features to learn accurate view-specific embeddings of each view [54].

For each view graph, the inputs to EGAT are *X*^0^, *E*^0^, and *Y*, where *X*^0^ is the feature matrix learned from gene expression (*X*) by a negative binomial distribution self-encoder framework, and *E*^0^ is an adjacency matrix (*E^m^* ∈ *R*^*n* × *n*^) of the *m*th graph *G^m^*, *m* ∈ {1, 2, 3}, and *Y* consists of the labels of each spot. Edge feature matrices are normalized into doubly stochastic matrices in the EGAT model, which has been recently demonstrated for graph edge denoising. Normalized features *E* are produced as

$$\begin{array}{c} {\tilde{E}}_{\mathrm{ijp}}=\frac{{\hat{E}}_{\mathrm{ijp}}}{\Sigma_{k=1}^{N}{\hat{E}}_{\mathrm{ikp}}},\\ E_{\mathrm{ijp}}=\Sigma_{k=1}^{N}\frac{{\tilde{E}}_{\mathrm{ikp}}{\tilde{E}}_{\mathrm{jkp}}}{\Sigma_{v=1}^{N}{\hat{E}}_{\mathrm{vkp}}} \end{array}$$

where $\hat{E}$ consists of the raw edge features, and all its elements are positive.

An attention mechanism is proposed for the problem of multidimensional positive real-valued edge features. Feature vector $X_{i∙}^{l}$ is aggregated from the feature vectors of neighboring nodes of the *i*th node, i.e., $\left\{X_{j},\;j\in N_{i}\right\}$ in the new attention mechanism, where $N_{i}\;$ consists of the indices of its neighbors. It simultaneously incorporates the corresponding edge features. The aggregation operation is defined as

$$X^{l}=\sigma \left[\left({∥}_{p=1}^{P}\alpha_{..p}^{l}(X^{l-1},\;E_{..p}^{l-1})g^{l}(X^{l-1}\right)\right],$$

where *σ* is a nonlinear activation function; ∥ represents the concatenation operator; *α* is a function used to generate an *N* × *N* × *P* tensor, *α^l^* consists of attention coefficients, and $\alpha_{\mathrm{ijp}}^{l}$ is a function of $X_{i∙}^{l-1}$, $X_{i∙}^{l-1}$, and *E_ijp_* as the entry of *α^l^*; *α*_.. *p*_ is its *p* channel matrix slice; and *g* is a linear mapping of the node features from input space to output space, and the transformation is defined as

$$g^{l}\left(X^{l-1}\right)=W^{l}X^{l-1},$$

where *W^l^* is an *F^l^* × *F*^*l* − 1^ parameter matrix.

$$\alpha_{∙∙p}^{l}=\mathrm{DS}\left({\hat{\alpha }}_{∙∙p}^{l}\right),$$

$${\hat{\alpha }}_{\mathrm{ijp}}^{l}=f^{l}(X_{i\cdot }^{l-1},X_{j∙}^{l-1})E_{\mathrm{ijp}}^{l-1},$$

where *DS* is the doubly stochastic normalization operator, and *f^l^* is an attention function, defined as

$$f^{l}(X_{i\cdot }^{l-1},X_{j∙}^{l-1})=\exp\left\{L\left(a^{T}\left[WX_{i∙}^{l-1}∥X_{j∙}^{l-1}\right]\right)\right\},$$

where *L* is the LeakyReLU activation function, *W* is the mapping defined by *g^l^*(*X*^*l* − 1^) = *W^l^X*^*l* − 1^, and ∥ is the concatenation operation. The attention coefficients are used as new edge features of the next layer, i.e.,

$$E^{l}=\alpha^{l}.$$

Consequently, EGAT adapts edge features crossing the network layers.

The loss function of the EGAT model is

$$L_{\text{egat}}=L_{\mathrm{adj}\_\text{deco}}+8L_{\mathrm{adj}\_\text{auto}},$$

where *L*_*adj*_*auto*_ is the cross-entropy between the adjacency matrices *A* and *A*^′^, *E*^′^ is the decoder of the EGAT model and is defined as the inner product between the low-dimensional feature *R* and *R^T^*,

$$E^{\prime }=\text{sigmoid}\left(R∙R^{T}\right).$$

*L*_*adj*_*auto*_ is defined as

$$L_{\mathrm{adj}\_\mathrm{demo}}=-\frac{1}{N\times N}\sum_{i=1}^{N}\sum_{j=1}^{N}(e_{ij}\times \log(e_{ij})+(1-e_{ij})\times \log(1-e_{ij})$$

We process the lower-dimensional feature to obtain the spot class prediction. The prediction is

$$Y^{\prime }=\text{softmax}(R),$$

and *L*_*adj*_*auto*_ is defined as

$$L_{\mathrm{adj}\_\text{auto}}=\frac{1}{u}\sum_{l=1}^{u}\left(-\sum_{i=1}^{K}y_{i}\log\left(y_{i}^{\prime }\right)\right),$$

where *u* is the number of spots that include training sets, *K* is the number of classes of labels, *y_i_* is the true class, and $y_{i}^{\prime }$ is the predicted class.

### Global attention mechanism

The GAM retains the information of channel and spatial aspects by reducing and magnifying the global interactive representations, which enhances the cross-dimension interactions [55].

EGAT captures the node embedding of 3 views under weak supervision. We concatenate them as the input of GAM as well as the labels (*Y*). GAM learns low-dimensional features under weak supervision. Given the feature map *F*_1_ ∈ *R*^*C* × *H* × *W*^ as input, the intermediate state *F*_2_ and output *F*_3_ are defined as

$$\begin{array}{c} F_{2}=M_{c}\left(F_{1}\right)⊗F_{1},\\ F_{3}=M_{s}\left(F_{2}\right)⊗F_{2} \end{array}$$

where *M_C_* is the channel attention mapping, *M_S_* represents spatial attention mapping, and ⨂ represents element multiplication [56].

In the channel attention submodule (Fig. 1), we use a 3D perceptron, which retains information across 3 dimensions. To magnify the cross-dimensional channel-spatial dependences, we use a 2-layer multilayer perceptron with a hidden layer, which is an encoder–decoder structure with a reduction ratio *γ* [57].

The spatial attention submodule (Fig. 1) uses 2 convolutional layers to fuse spatial information. GAM uses group convolution with channel shuffle in ResNet50 to prevent a notable increase in parameters.

The low-dimensional feature is produced as

$$R=F_{3}[1]+F_{3}[2]+F_{3}[3],$$

where *F*_3_[*m*] represents the *m^th^* channel of output *F*_3_.

We process the lower-dimensional feature generated by GAM to obtain the spot class prediction, and the predictionis

$$Y^{\prime }=\text{softmax}(R).$$

The loss function of GAM is defined as

$$L_{\mathrm{GAM}}=\frac{1}{u}\sum_{l=1}^{u}\left(-\sum_{i=1}^{K}y_{i}\log\left(y_{i}^{\prime }\right)\right),$$

where *u* is the number of spots in the training set of the GAM model, *K* is the number of classes of labels, *y_i_* is the true class, and $y_{i}^{\prime }$ is the predicted class.

### Identifying 3D spatial domains by stMGATF

Most current SRT techniques profile gene expression patterns in the context of 2D tissue slices and ignore the 3D information in real-world SRT data. Considering that EGAT can exploit multidimensional edge information, stMGATF-3D exploits information from adjacent tissue slices to reduce the batch effect of slices. stMGATF can effectively identify 3D spatial domains by integrating adjacent points in 2D slices and adjacent slice information in 3D space. Different from using only 2D slice information, stMGATF-3D exploits the position information of neighbor slices to construct SLGs for the extraction of low-dimensional features. We use gene expression features as node features and process the physical location (*s_ix_*, *s_iy_*, *s_iz_*) to form 4-dimensional edge features to construct a SLG, with edge features defined as

$$\begin{array}{c} e_{\mathrm{ij}}^{1}=\frac{\left(s_{ix},s_{iy})\cdot (s_{jx},s_{jy}\right)}{\sqrt{{\left(s_{\mathrm{ix}}-s_{\mathrm{jx}}\right)}^{2}+{\left(s_{iy}-s_{jy}\right)}^{2}}},\\ {\hat{e}}_{\mathrm{ij}}^{2}=\sqrt{{\left(s_{\mathrm{ix}}-s_{\mathrm{jx}}\right)}^{2}+{\left(s_{iy}-s_{jy}\right)}^{2}},\\ e_{\mathrm{ij}}^{2}=\left\{\begin{array}{c} 1,{\hat{e}}_{\mathrm{ij}}^{2}\leq \lambda \\ 0,{\hat{e}}_{\mathrm{ij}}^{2}>\lambda , \end{array}\right.\\ {\hat{e}}_{\mathrm{ij}}^{3}=|s_{\mathrm{iy}}-s_{\mathrm{jy}}|,\\ e_{\mathrm{ij}}^{3}=\left\{\begin{array}{c} 1,{\hat{e}}_{\mathrm{ij}}^{3}\leq \mu \\ 0,{\hat{e}}_{\mathrm{ij}}^{3}>\mu , \end{array}\right.\\ {\hat{e}}_{\mathrm{ij}}^{4}=|s_{\mathrm{iz}}-s_{jz}|,\\ e_{\mathrm{ij}}^{4}=\left\{\begin{array}{c} 1,{\hat{e}}_{\mathrm{ij}}^{4}\leq \xi \\ 0,{\hat{e}}_{\mathrm{ij}}^{4}>\xi \end{array}\right. \end{array}$$

where || indicates the absolute value operation; μ, λ, and *ξ* are hyperparameters; and $e_{\mathrm{ij}}^{1}$, $e_{\mathrm{ij}}^{2}$, $e_{\mathrm{ij}}^{3}$, and $e_{\mathrm{ij}}^{4}$ represent the 3D features of edges.

### The implementation details of stMGATF on different datasets

In this study, we utilized various datasets to verify the effectiveness of the stMGATF. The datasets included DLPFC [11], 10x Visium spatial transcriptomics data of human breast cancer [6] and STARmap [21], both of which had manually annotated labels. Additionally, we used the Slide-seq dataset of mouse hippocampus, which lacked manual annotations. To overcome this limitation, we associated the coordinates of the cells in this dataset with the positional information provided by Rodriques et al. [20] and obtained the annotated information of the tissue structure from the Allen Brain Atlas at a similar location. We used these annotated labels to extract potential embeddings during training.

The implementation involved several steps for each dataset:

Step 1: The input consisted of a gene expression matrix, histological images, spot location information, and a conditional degree matrix. We employed the SimCLRv2 method to learn visual features from the histological images and used an autoencoder framework to extract gene expression features and conditional degree matrix features. The output of this step was gene expression features, visual features of histological images, spot locations, and conditional degree matrix features.

Step 2: In this step, we constructed HSG, SLG, and GAG using the gene expression features, visual features of histological images, physical locations, and gene associations. We then employed EGAT to obtain potential embeddings for each view. The output of this step was low-dimensional potential embeddings from each view.

Step 3: We adaptively integrated the 3 views to obtain a low-dimensional representation using the GAM method. The input to this step was the low-dimensional potential embeddings extracted from each of the 3 views, and the output was the final fused low-dimensional features.

### Parameter analysis

Regarding the parameters used in stMGATF, there are 2 critical parameters, μ and λ, involved in constructing SLGs. We used gene expression features as node features and processed the physical location (*s_ix_*, *s_iy_*) to form 3D edge features and construct a SLG. The edge features were defined as follows:

$$\begin{array}{c} e_{\mathrm{ij}}^{1}=\frac{\left(s_{ix},s_{iy})\cdot (s_{jx},s_{jy}\right)}{\sqrt{{\left(s_{\mathrm{ix}}-s_{\mathrm{jx}}\right)}^{2}+{\left(s_{iy}-s_{jy}\right)}^{2}}},\\ {\hat{e}}_{\mathrm{ij}}^{2}=\sqrt{{\left(s_{\mathrm{ix}}-s_{\mathrm{jx}}\right)}^{2}+{\left(s_{iy}-s_{jy}\right)}^{2}},\\ e_{\mathrm{ij}}^{2}=\left\{\begin{array}{c} 1,{\hat{e}}_{\mathrm{ij}}^{2}\leq \lambda \\ 0,{\hat{e}}_{\mathrm{ij}}^{2}>\lambda , \end{array}\right.\\ {\hat{e}}_{\mathrm{ij}}^{3}=|s_{\mathrm{iy}}-s_{\mathrm{jy}}|,\\ e_{\mathrm{ij}}^{3}=\left\{\begin{array}{c} 1,{\hat{e}}_{\mathrm{ij}}^{3}\leq \mu \\ 0,{\hat{e}}_{\mathrm{ij}}^{3}>\mu , \end{array}\right. \end{array}$$

where || indicates the absolute value operation, μ and λ are hyperparameters, and $e_{\mathrm{ij}}^{1},e_{\mathrm{ij}}^{2},\text{and}\;e_{\mathrm{ij}}^{3}$ represent the 3D features of edges.

We investigated the influence of hyperparameters μ and λ on a dataset comprising 151,673 slices (Fig. S1). Figure S1 visually presents the results of the ASW [10] in a 2D representation. By analyzing Fig. S1, we observed that the choice of parameter values for μ and λ plays a crucial role in the proposed model. Notably, the optimal clustering outcome was achieved when μ was set to 10 and λ to 200, using the entire dataset of 151,673 slices. This finding highlights the significance of carefully selecting the threshold for constructing the edges of the graph. In our study, we adopted the threshold (10,200) to define the edges of the SLG. As for other datasets, μ and λ can be adjusted.

### Clustering

We conducted a clustering analysis using k-means algorithm on the extracted potential embeddings to obtain the spot class prediction. For k-means clustering, the number of clusters is predetermined. In situations where the number of classes is unknown, the Davies–Bouldin Index (DBI) can serve as a useful reference. The DBI quantifies the ratio between cluster closeness and separation, where a smaller DBI value corresponds to smaller intraclass distances and larger interclass distances [58]. In our study, we initially predefined the range of the number of clusters. Subsequently, we calculated the DBI values for each clustering result and selected the number of clusters associated with the smallest DBI value.

### UMAP visualization on STARmap dataset

For stMGATF, a low-dimensional latent embedding extracted by fusion of 2 views, stMGATF-SLG and stMGATF-GAG, was utilized as an input to UMAP on the STARmap dataset [21]. For STAGATE, we exploited the STAGATE 1.0.0 documentation as a reference and the low-dimensional features extracted by STAGATE as UMAP input according to Tutorial 9.

### The implementation of survival analysis

To examine the prognostic significance of SDGs, we utilize breast cancer data. Specifically, we acquire RNA-seq data along with clinicopathologic and survival data of TCGA BRCA from the UCSC Xena database.

Subsequently, we categorize the BRCA samples into high and low groups based on the median gene expression and pseudo-expression. By employing the Kaplan–Meier method, we generate survival curves for each group. To compare the differences in survival, we employ the log-rank test, a 2-sided statistical test. We consider *P* values less than 0.05 as statistically significant.

## Data Availability

All data analyzed in this paper are available in publicly available SRT datasets. Specifically, the 12 slices of human DLPFC dataset [11] are available from spatialLIBD (http://spatial.libd.org/spatialLIBD/).The 10x Visium human breast cancer dataset can be downloaded from https://support.10xgenomics.com/spatial-gene-expression/datasets/1.0.0/V1_Breast_Cancer_Block_A_Section_1. The annotation file can be found on the SEDR website: https://github.com/JinmiaoChenLab/SEDR_analyses/tree/master/data/BRCA1. Slide-seq datasets [20] are available at https://portals.broadinstitute.org/single_cell/study/slide-seq-study. The mouse visual cortex STARmap data [21] is from https://www.dropbox.com/sh/f7ebheru1lbz91s/AADm6D54GSEFXB1feRy6OSASa/visual_1020/20180505_BY3_1kgenes?dl=0&subfolder_nav_tracking=1. The bulk RNA-seq and clinical data from the TCGA database are at the Xena platform (https://xenabrowser.net/datapages/). **Code availability:** stMGATF is implemented based on python 3.6.12 and R 4.1.1. Other tools and packages used in the data analysis include the following: numpy 1.19.2, pandas 1.1.5, scipy 1.5.2, scikit-learn 0.23.3, torch 1.6.0, tqdm 4.55.0, scanpy1.6.0, PIL 9.1.0, seaborn 0.11.2, sklearn 1.0.2, matplotlib 3.5.2, glob2, anndata 0.8.0, argparse 1.1, json 2.0.9, Seurat v3, ggplot2 3.3.5, and monocle 2.10.1. The code used for this paper is available on GitHub (https://github.com/liying-1028/stMGATF).
