# Supplementary material for: Revealing Tissue Heterogeneity and Spatial Dark Genes from Spatially Resolved Transcriptomics by Multiview Graph Networks
Source: Research (Wash D C). 2023 Sep 20;6:0228. doi: 10.34133/research.0228 (PMC10511271; doi:10.34133/research.0228)
Supplement: Supplementary 1 — Figs. S1 to S17 Tables S1 to S3 [file research.0228.f1.docx]

**Supplementary Materials for**

**Revealing tissue heterogeneity and** **spatial dark-genes from spatially resolved transcriptomics by multi-view graph networks**

**Authors** Ying Li^1†^, Yuejing Lu^1†^, Chen Kang^1†^, Peiluan Li^1^,^2^*, Luonan Chen^3,4,5^*

**Affiliations**

^1^ School of Mathematics and Statistics, Henan University of Science and Technology, Luoyang 471023, China.

^2^ Longmen Laboratory, Luoyang, Henan, 471003, China.

^3^ Key Laboratory of Systems Biology, Institute of Biochemistry and Cell Biology, Center for Excellence in Molecular Cell Science, Chinese Academy of Sciences, Shanghai, 201100, China.

^4^ Key Laboratory of Systems Health Science of Zhejiang Province, Hangzhou Institute for Advanced Study, University of Chinese Academy of Sciences, Hangzhou, 310000, China.

^5^ School of Life Science and Technology, ShanghaiTech University, Shanghai, 201100, China

*Address correspondence to: Luonan Chen; [lnchen@sibs.ac.cn](mailto:lnchen@sibs.ac.cn) and Peiluan Li; [9902639@haust.edu.cn](mailto:9902639@haust.edu.cn)

† These authors contributed equally to this work as the first authors.

**
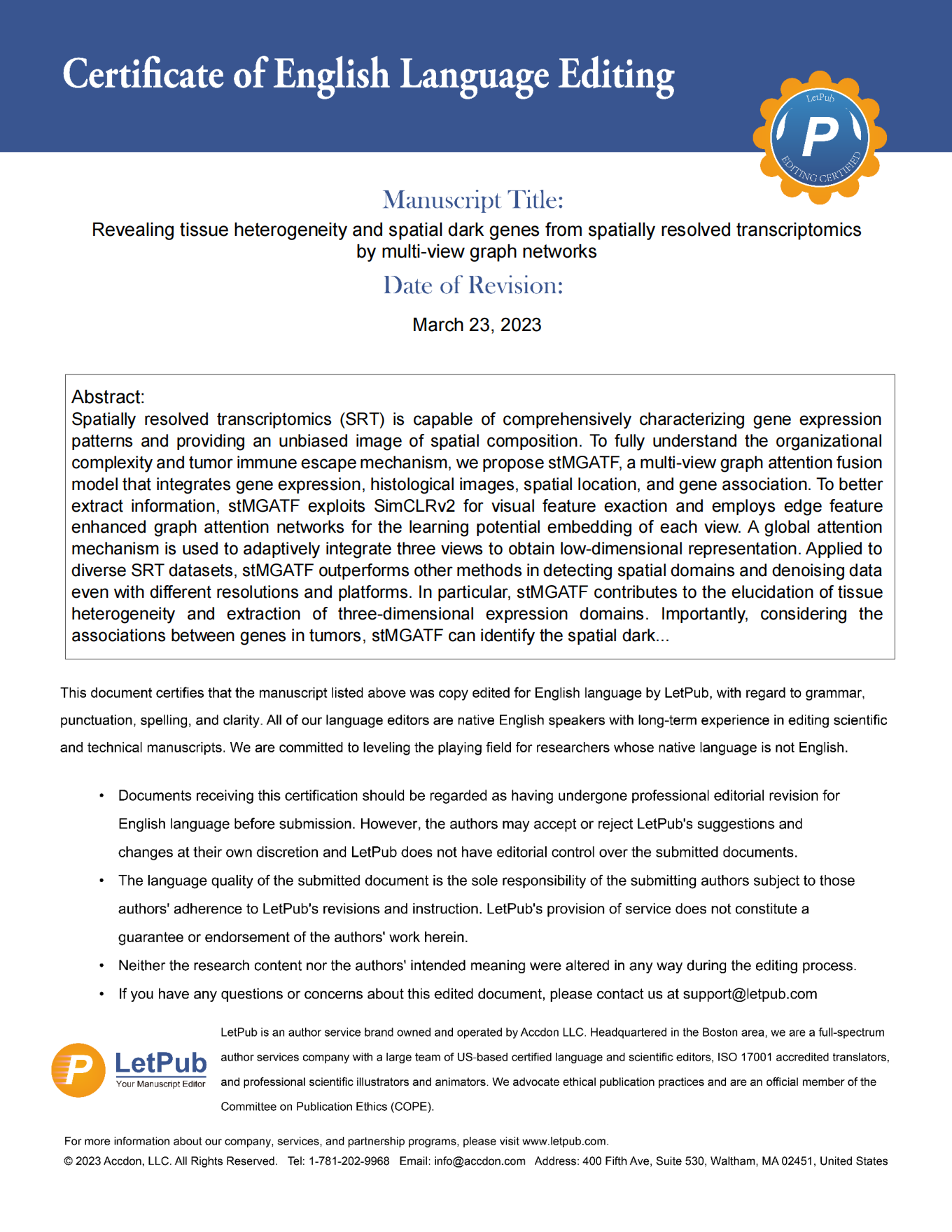
**

**materials and methods**

**Comparison with other spatial domain detection methods.**

We use the default parameter settings for all methods and the same number of clusters in clustering.

**Seurat:**

We used Seurat 's louvain algorithm for clustering^1^. The data preprocessing of Seurat is to log-normalize the expression in these spots and select the top 2,000 HVGs. The shared nearest neighbors graph was constructed with seurat::FindNeighbors(), and clustering was done via seurat::FindClusters(). We tuned the resolution parameter manually to obtain the specified number of clusters for each sample. For example, the resolution parameter is set to 0.32 in section 151673 of DLPFC.

**BayesSpace**^2^**:**

We used spatialPreprocess() functions to log-normalize the expression in these spots, then the top 15 PCs on the log-normalized expression of the top 2,000 highly variable genes in each dataset. We used the qTune() and qPlot() functions to help choose ‘q’, which is around the elbow of the plot. The seed parameter was set as 149 in the DLPFC section 151673. BayesSpace was applied to the DLPFC section 151673 according to its defaulted parameters.

**Giotto**^3^**:**

We applied Giotto to DLPFC dataset to detect spatial domain. At first, we used filterGiotto() function to filter genes and cells with expression_threshold = 1, and used normalizeGiotto() function to normalize expression in these spots with scalefactor = 6000. Then Giotto::doHMRF() was used to obtain spatial domains from spatially expressed genes and createSpatialNetwork() function was used to construct spatial network with k=5 and maximum_distance_knn=400.

**SpaGCN**^4^**:**

SpaGCN takes spatial gene expression and histology image data (when available) as input. The gene expression values in each spot are normalized such that the UMI count for each gene is divided by the total UMI count across all genes in a given spot, multiplied by 10,000, and then transformed to a natural log scale. We adopted SpaGCN on the DLPFC dataset using its recommended parameters in the package vignette.

**stLearn**^5^**:**

stLearn using SME normalized the raw counts of all genes. The SME normalized matrix is the input for linear PCA dimensionality reduction, followed by UMAP embedding, and k-nearest neighbor (kNN) graph construction. stLearn was applied to the DLPFC section 151673 dataset according to its recommended parameters.

**STAGATE**^6^**:**

STAGATE was applied to the DLPFC dataset. The spatial network was first constructed using the STAGATE. Cal_Spatial_Net() function with rad_cutoff=150 and the embedding is trained using the STAGATE.train_STAGATE() with alpha=0,epoch=500, and lr=0.0001.

**stMVC**^7^**:**

Through the semi-supervised learning of 70% of the annotation, the parameter is default.

For preprocessing raw data and manual cell segmentation:

batch_size_I: defines the batch size for training SimCLR model. The default value is 128. You can modify it based on your memory size. The larger the parameter, the less time. max_epoch_I: defines the max iteration for training SimCLR model. The default value is 500. You can modify it. The smaller the parameter, the less time.

For stMVC model:

lr_T1 for HSG, lr_T2 for SLG, lr_T3 for collaborative learning: defines learning rate parameters for learning view-specific representations by single-view graph and robust representations by multi-view graph, i.e. The default value of the three parameters is 0.002. max_epoch_T: defines the max iteration for training view-specific graph or multi-view graphs. The default value is 500. You can modify it. The larger the parameter, the more time. beta_pa: defines the penalty for the knowledge transfer from robust representations to view-specific representations. The default value is 8. knn: defines the K-nearest similarity spots for each spot to construct HSG or SLG. The default value is 7 where the K-nearest spots for a spot include itself. latent_T1 and latent_T2 define the dimension of two layers of GAT for SGATE model. Here, the default value of the DLPFC and IDC datasets is 25 and 10, 32 and 16, respectively. fusion_type: definies the multi-view graph fusion types. The default value of the DLPFC and IDC datasets is 25 and 10, 32 and 16, respectively. fusion_type: definies the multi-view graph fusion types. The default value is Attention, using Mean to perform stMVC-M.

**Details of datasets**

**DLPFC**

DLPFC is a ST dataset containing 12 samples, each of which consists of up to six cortical layers and the white matter^8^. As a result, every sample includes 5 or 7 annotated layers and a small set of unlabeled plots. This is a benchmark dataset utilized in recent approaches for evaluating the performance of clustering.

Dataset link: <http://research.libd.org/globus/jhpce_HumanPilot10x/index.html>.

**10x Visium spatial transcriptomics data of human breast cancer**

The annotation of 10x Visium spatial transcriptomics data of human breast cancer annotation is provided by SEDR. the tissue is segmented to 20 areas within four main morphotypes, which are: Ductal Carcinoma in Situ/Lobular Carcinoma in Situ (DCIS/LCIS), healthy tissue (Healthy), Invasive Ductal Carcinoma (IDC), and tumor surrounding regions with low features of malignancy (Tumor edge).

Dataset link:

[https://support.10xgenomics.com/spatial-gene-expression/datasets/1.0.0/V1_Breast_ Cancer_Block_A_Section_1](https://support.10xgenomics.com/spatial-gene-expression/datasets/1.0.0/V1_Breast_%20Cancer_Block_A_Section_1)

The annotation file for 10x Visium spatial transcriptomics data of human breast cancer is from: <https://github.com/JinmiaoChenLab/SEDR_analyses/tree/master/data/BRCA1>

**STARmap**

The STARmap dataset that has single-cell resolution^7^. This dataset was generated from mouse visual cortex that spans from hippocampus to corpus callosum, and the six neocortical layers. In total, 1,020 genes were measured in 1,207 cells that include non-neuronal cells, excitatory and inhibitory neurons.

Dataset link:

<https://www.dropbox.com/sh/f7ebheru1lbz91s/AADm6D54GSEFXB1feRy6OSASa/visual_1020/20180505_BY3_1kgenes?dl=0&subfolder_nav_tracking=1>

**Slide-seq**

Samuel G. Rodriques et al sequenced pucks capturing 66 sagittal tissue sections from a single dorsal mouse hippocampus^9^. We applied STAGATE onto a pseudo-3D ST data constructed by aligning the spots of the “cord-like” structure in seven hippocampus sections profiled by Slide-seq.

Dataset link:

[https://portals.broadinstitute.org/single_cell/ study/slide-seq-study](https://portals.broadinstitute.org/single_cell/%20study/slide-seq-study).

**Metrics**

**Adjusted Rand Index (ARI)**

The ARI is the corrected-for-chance version of the Rand index (RI), which is a measure of the similarity between two clusters. ARI can be obtained by:

$$ARI=\frac{\max\left( RI \right)-E(RI)}{RI-E(RI)},$$

where $RI$ is:

$$RI=\frac{TP+TN}{TP+FP+TN+FN}$$

**Average Silhouette Width (ASW)**

The Silhouette value is used to evaluate the similarity of a destination to other clusters in the cluster where the destination is located^10^. The range of Silhouette value is from -1 to +1. The value reflects the matching relationship between the target and their own clusters, a high value indicates a high matching relationship with other clusters, conversely, a low value indicates a low matching relationship with other clusters. If the value is high, then the clustering result is better, if it is small or negative, then it may be caused by too many or too few clusters.

Suppose the dataset has been divided into many classes by the clustering algorithm and for the objective $i$ there are $i\in C_{i}$,obtaining:

$$a\left( i \right)=\frac{1}{|C_{i}-1|}\sum_{j\in C_{i},i\neq j} d(i,j)$$

where $a\left( i \right)$ represents the average distance between$i$ and other targets between the same cluster. $d(i,j)$ is the distance of targets $i$ and $j$ in cluster $C_{i}$.

**Supplementary Figures**

**Supplementary Figure 1.** The DBI values for different number of clusters of predicting results on 151673 slices.

**Supplementary Figure 2**. The ARI values for different methods of predicting results on 151673 slices.

**Supplementary Figure 3.** Analysis of different hyperparameters ($\mu,\lambda$) with ASW on 151673 slices.

**Supplementary Figure 4.** Spatial domains were detected by stMGATF-SLG, stMGATF-GAG, stMGATF and manual annotation as a comparison, on 12 slices of the DLPFC dataset.

**Supplementary Figure 5.** Scatter plot of the two-dimensional UMAP extracted from the latent features by stMGATF-SLG, stMGATF-GAG, stMGATF, on 12 slices of the human DLPFC dataset.

**Supplementary Figure 6.** Spatial expression of layer-specific genes: *MOBP*, *KRT17*, *PCP4*, *PVALB*, *ENC1*,*CCK* and for slice 151673 data denoised by stMGATF, respectively.

**Supplementary Figure 7.** Gene function enrichment analysis of genes differentially expressed between stMGATF clusters 11 and 14.

**Supplementary Figure 8.** Distribution of cells in each cluster on the pseudo-time trajectory.

**Supplementary Figure 9.** Data analysis in breast cancer sample.

**Supplementary Figure 10.** stMGATF-2D uncovers the spatial domain-specific SVGs on the onto a pseudo-3D ST data constructed by aligning the spots of the "cord-like" structure in seven hippocampus sections profiled by Slide-seq.

**Supplementary Figure 11.** stMGATF-3D uncovers the spatial domain-specific SVGs on the onto a pseudo-3D ST data constructed by aligning the spots of the "cord-like" structure in seven hippocampus sections profiled by Slide-seq.

**Supplementary Figure 12.** Spatial expression patterns of SVGs detected by stMGATF.

**Supplementary Figure 13.** KEGG functional enrichment analysis of spatial dark genes (SDGs). The significance threshold is 0.05.

**Supplementary Figure 14.** Co-expression network of the top ten upstream transcription factors identified by SDGs.

**Supplementary Figure 15.** Networks showed the relation of regulation from four ten upstream transcription factors to SDGs.

**Supplementary Figure 16.** Comparison of hub upstream TF PRRX2 survival curves in gene expression and network degree pseudo-expression constructed by stMGATF from RNA-seq data of breast cancer in TCGA database.

**Supplementary Figure 17.** Correlation scatter plot of ZNF469 and breast cancer metastasis related TFs expression in BRCA with lateral histogram.

**Supplementary Tables**

**Supplementary Table 1.** The result of 10-fold cross-validation.

**Supplementary Table 2.** KEGG pathway analysis of SPVGs.

**Supplementary Table 3.** GO enrichment analysis of transcription factors.


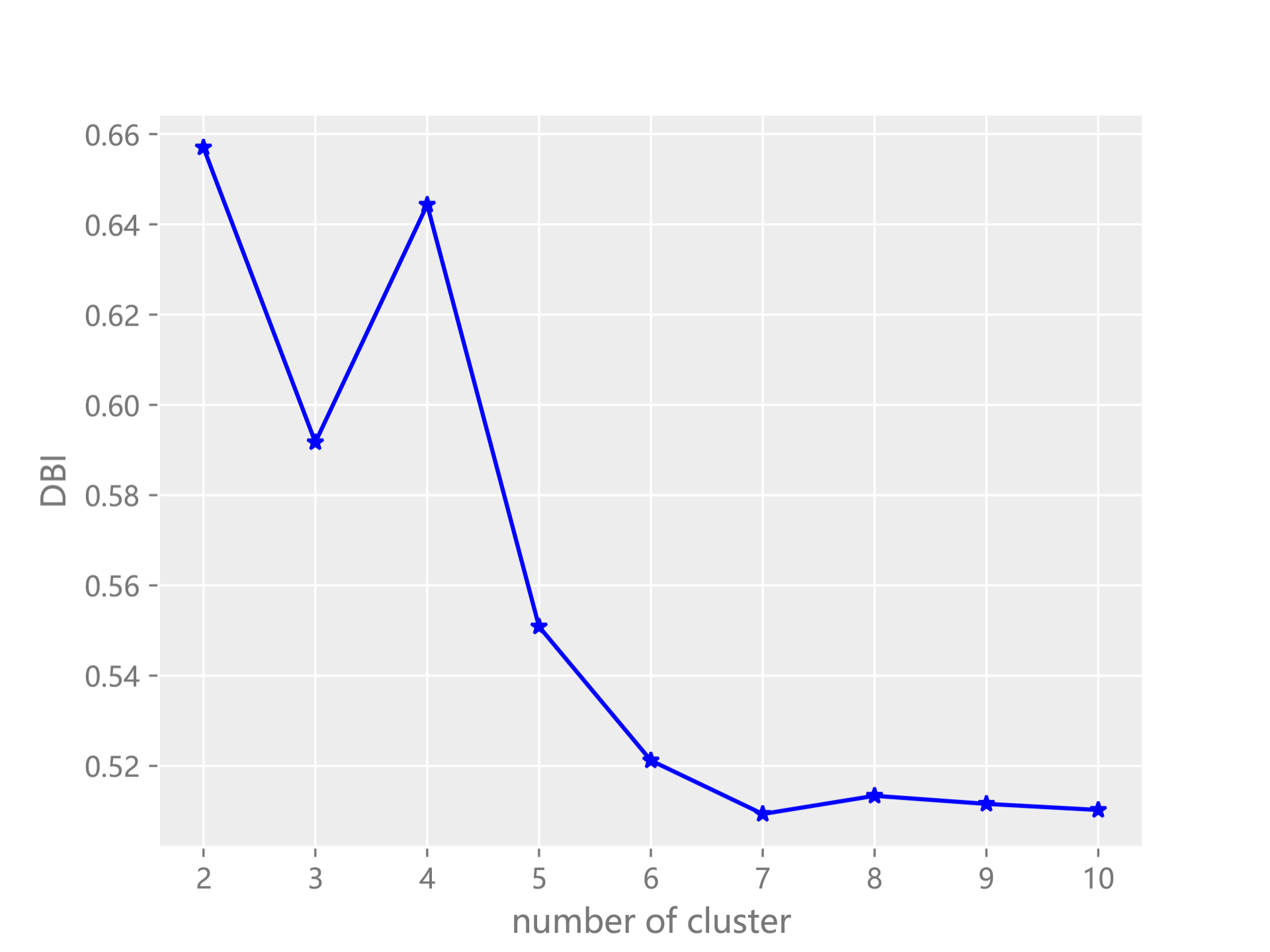


**Supplementary Figure 1.** The DBI values for different number of clusters of predicting results on 151673 slices.


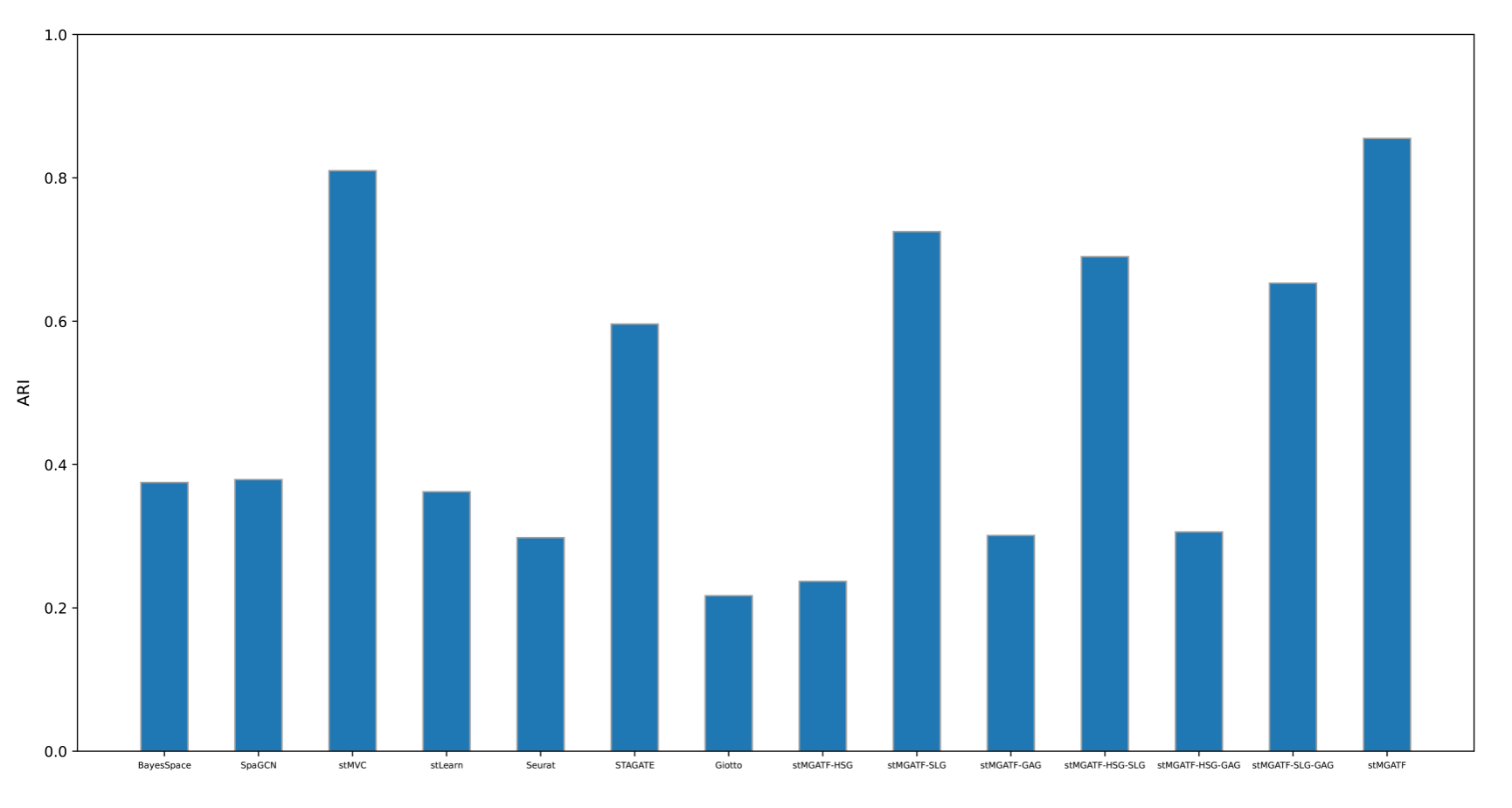


**Supplementary Figure 2**. The ARI values for different methods of predicting results.


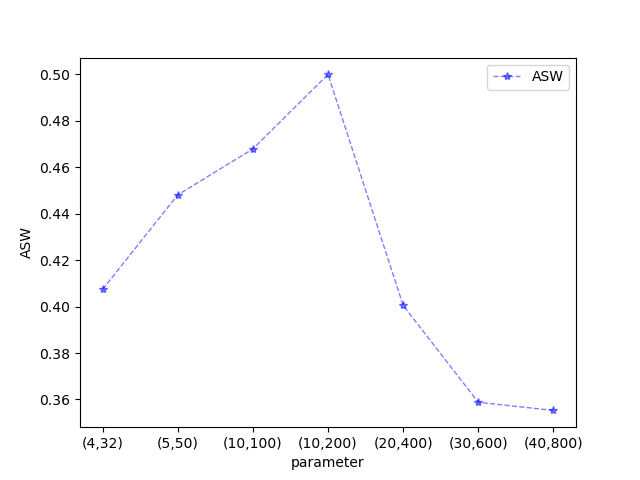


**Supplementary Figure 3.** Analysis of different hyperparameters ($\mu,\lambda$) with ASW on 151673 slices. We illustrate the results these hyperparameters in a 2D figure manner.


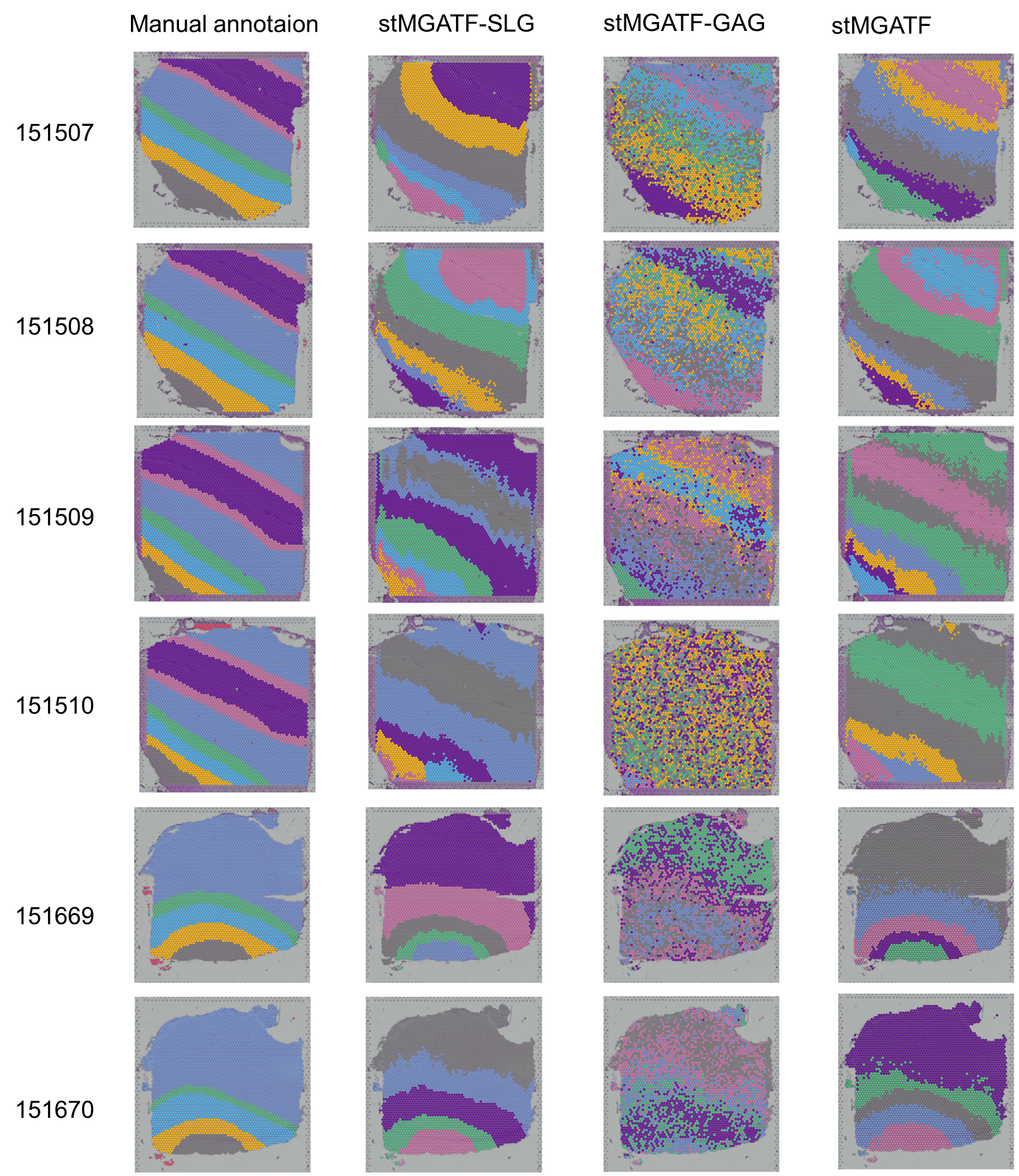


**See next page**


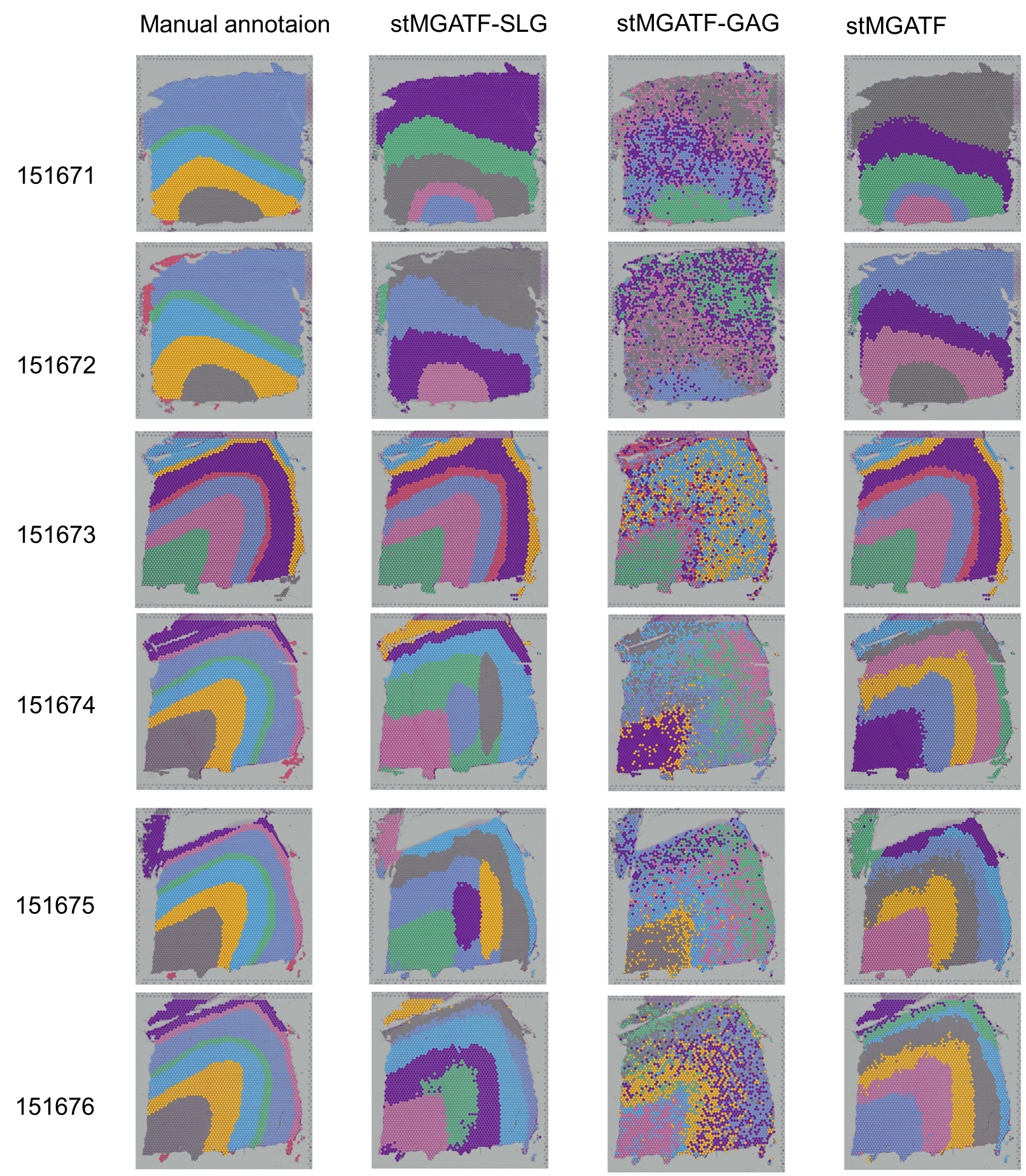


**Supplementary Figure 4.** Spatial domains were detected by stMGATF-SLG, stMGATF-GAG, stMGATF and manual annotation as a comparison, on 12 slices of the DLPFC dataset. Source data are provided as a Source Data file.


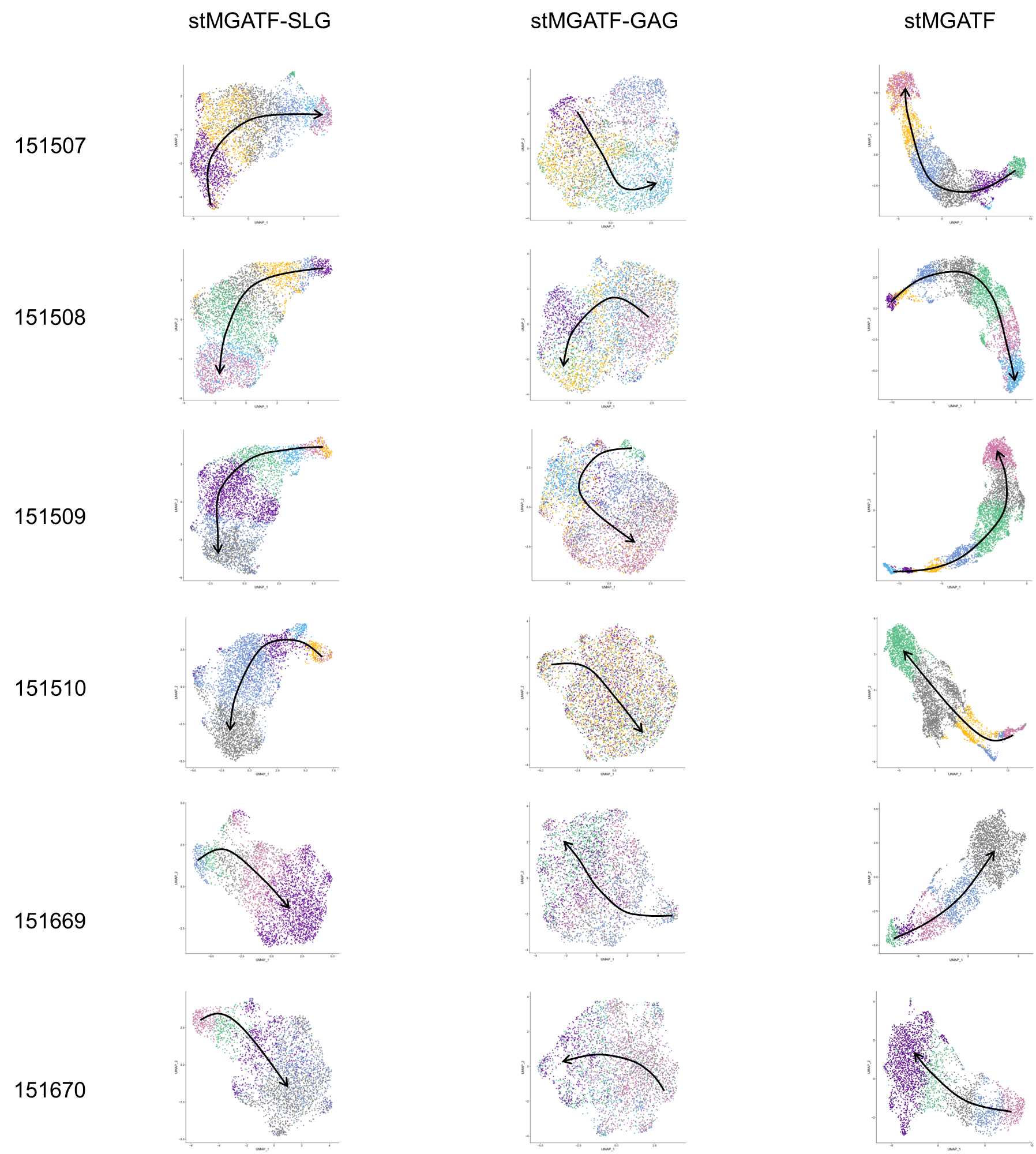


**See next page**


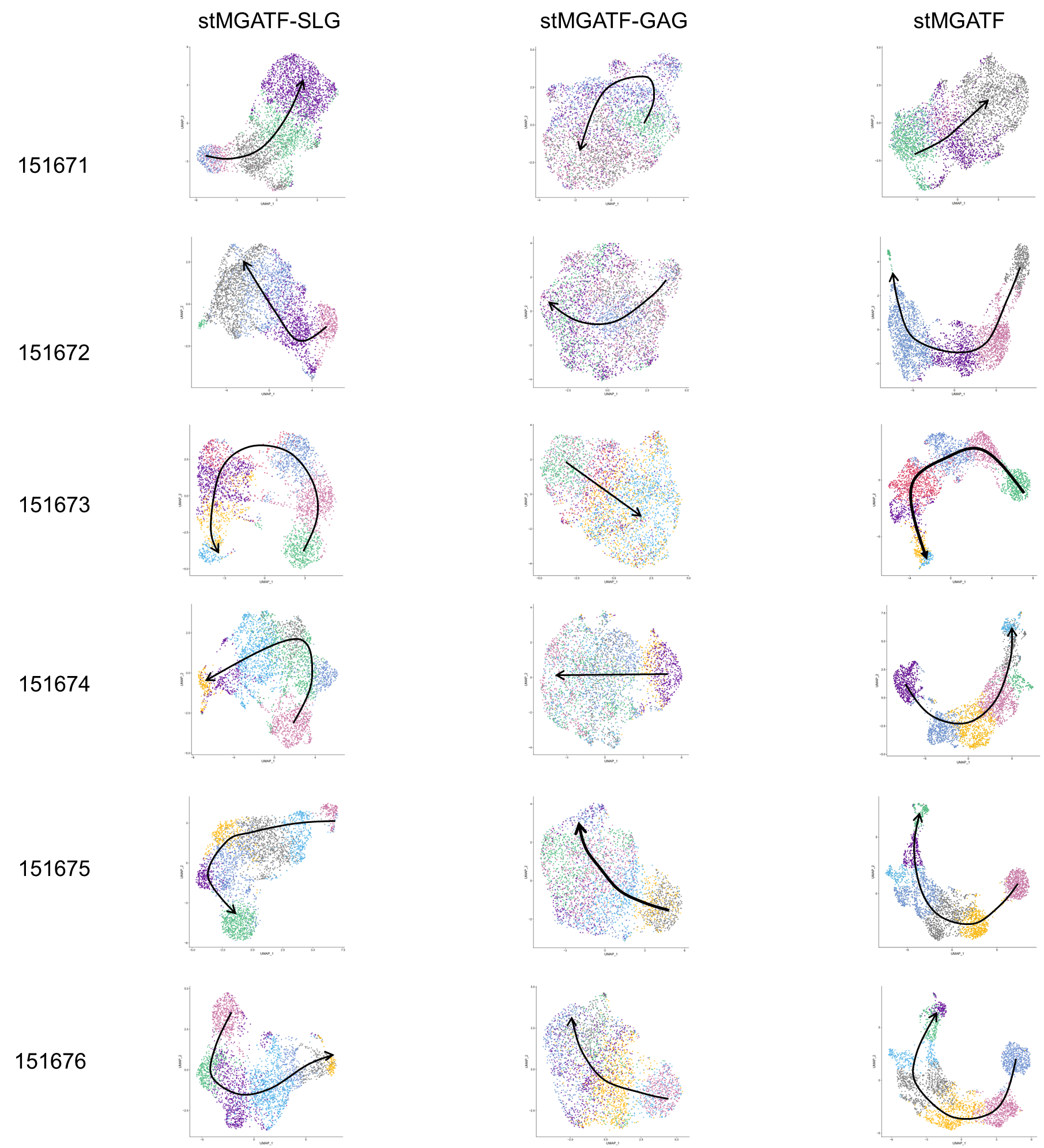


**Supplementary Figure 5.** Scatter plot of the two-dimensional UMAP extracted from the latent features by stMGATF-SLG, stMGATF-GAG, stMGATF, on 12 slices of the human DLPFC dataset. For each method on each slice, the predicted clusters and their colors are the same as Supplementary Fig.2. Source data are provided as a Source Data file.


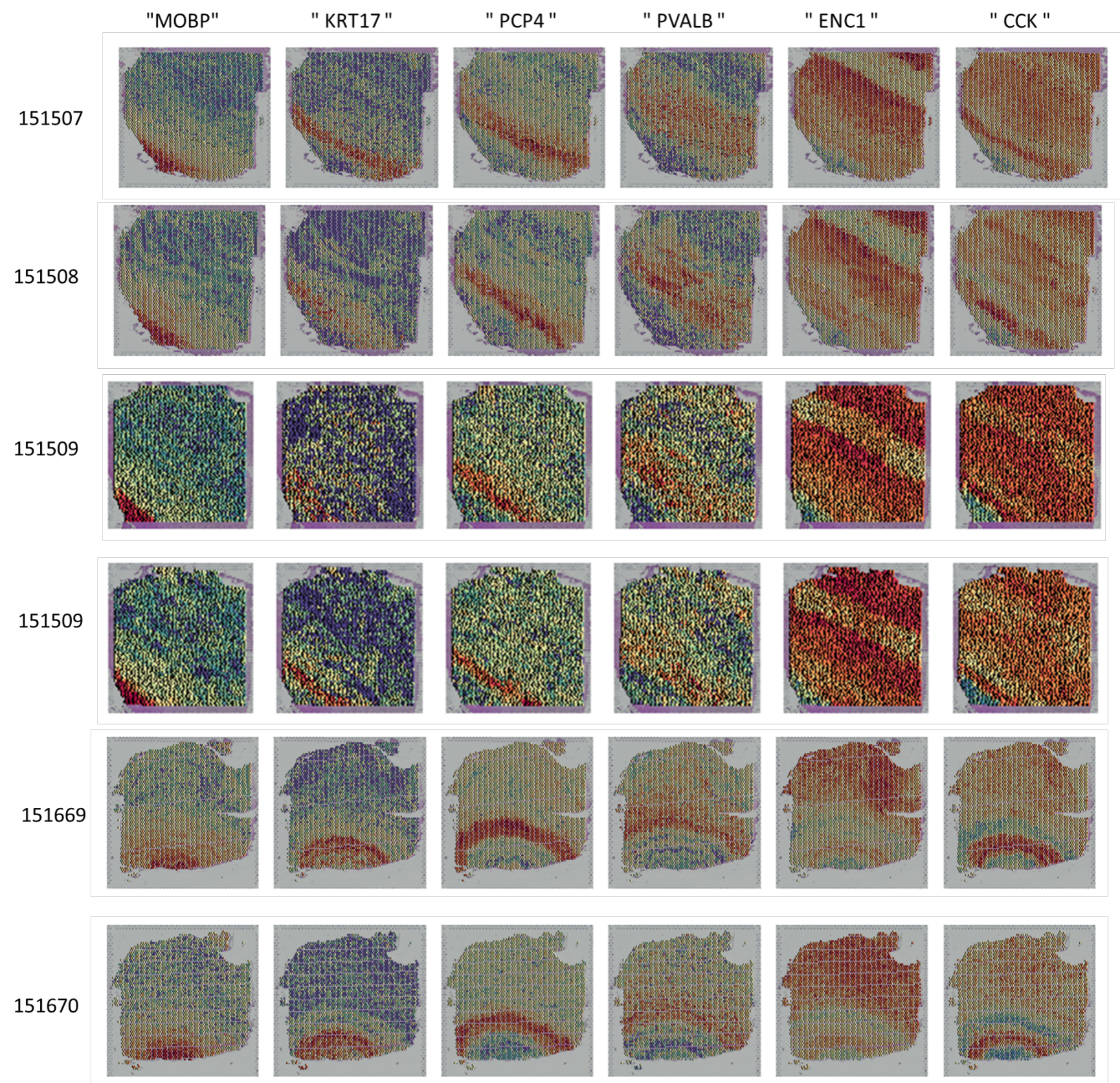


**See next page**


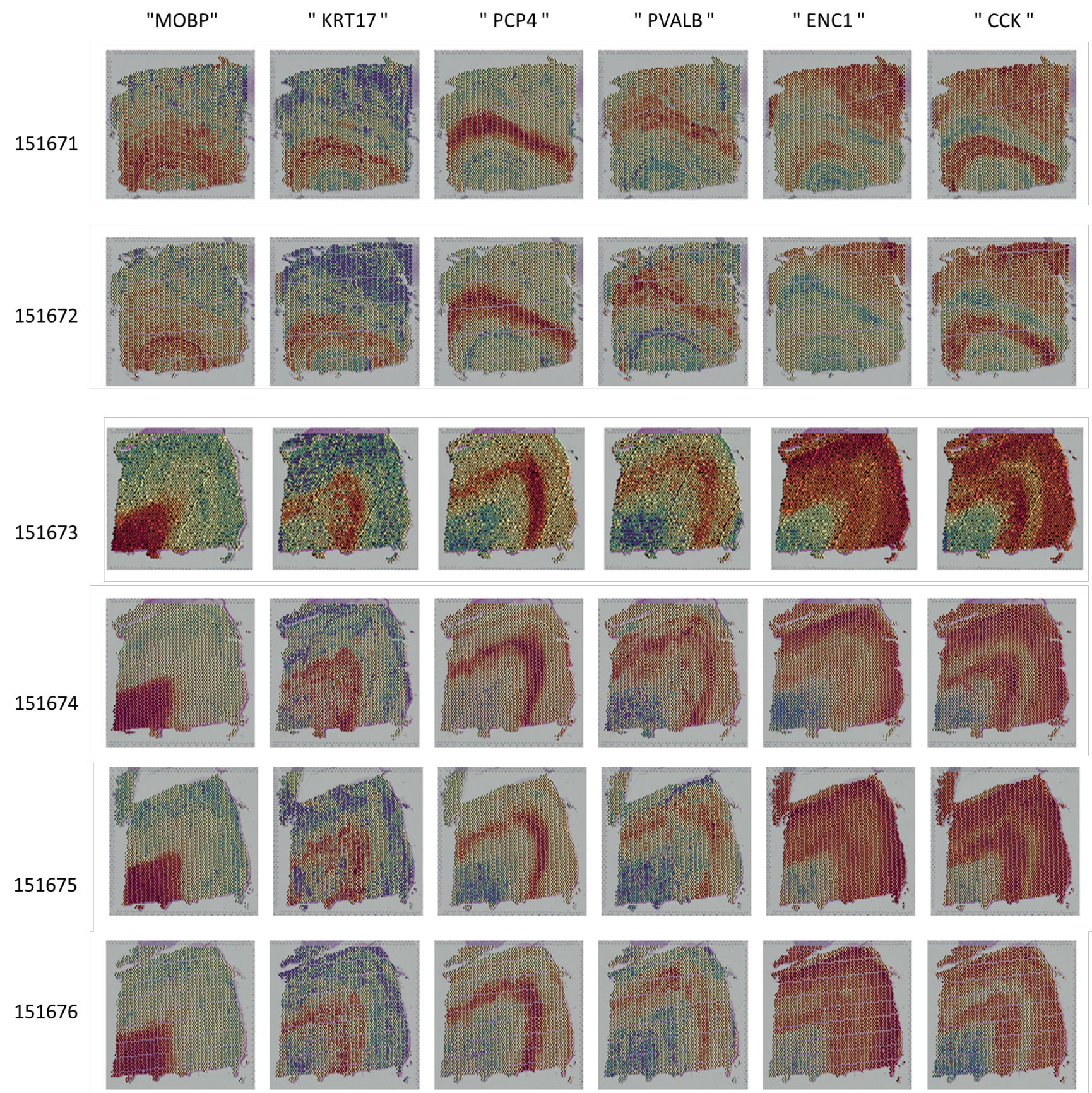


**Supplementary Figure 6.** Spatial expression of layer-specific genes^8^: *MOBP*, *KRT17*, *PCP4*, *PVALB*, *ENC1*,*CCK*, and for slice 151673 data denoised by stMGATF, respectively. Source data are provided as a Source Data file.


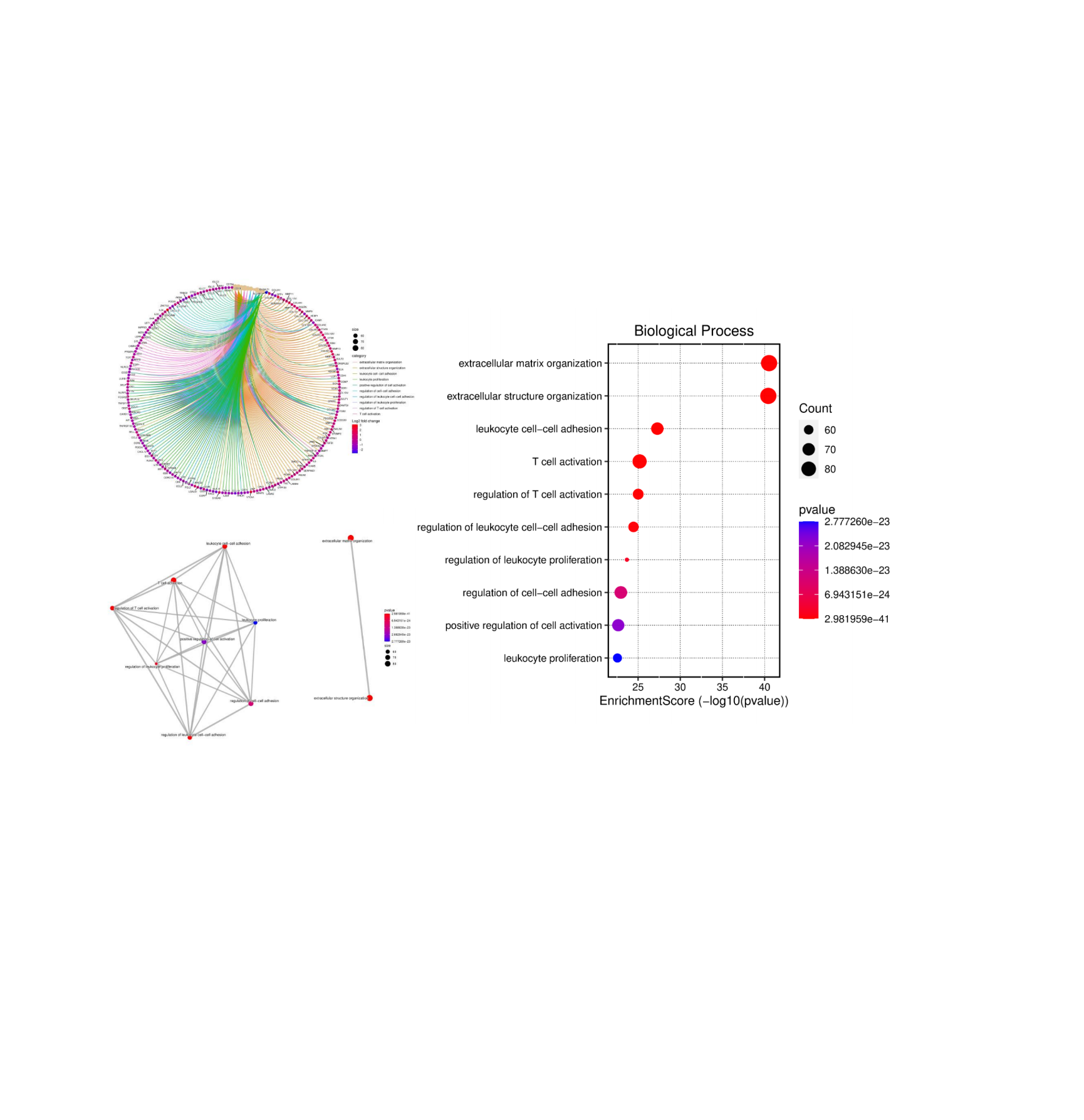


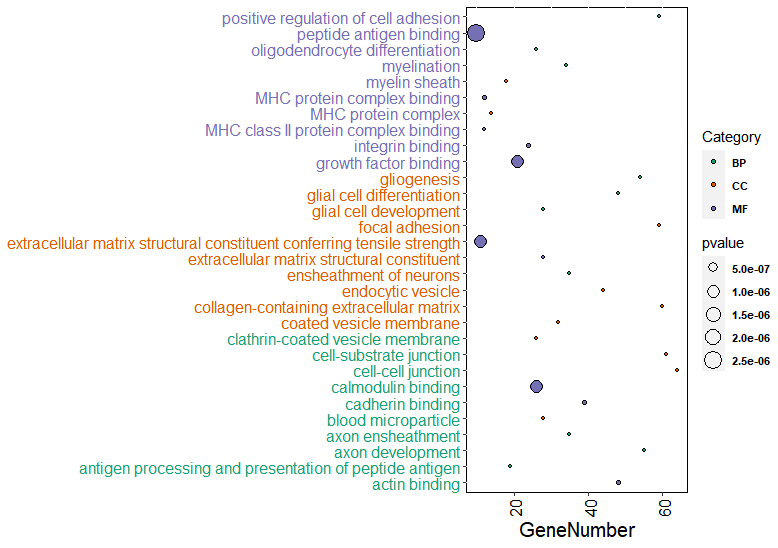

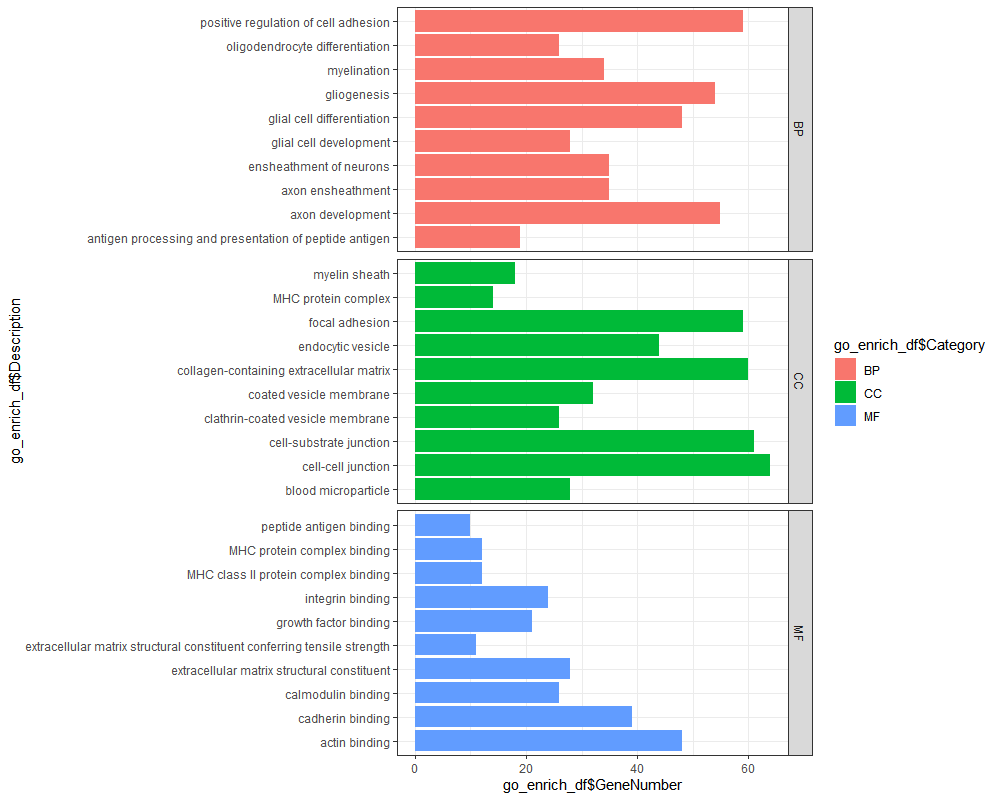


**Supplementary Figure 7.** Gene function enrichment analysis of SPVGs between stMGATF clusters 11 and 14.


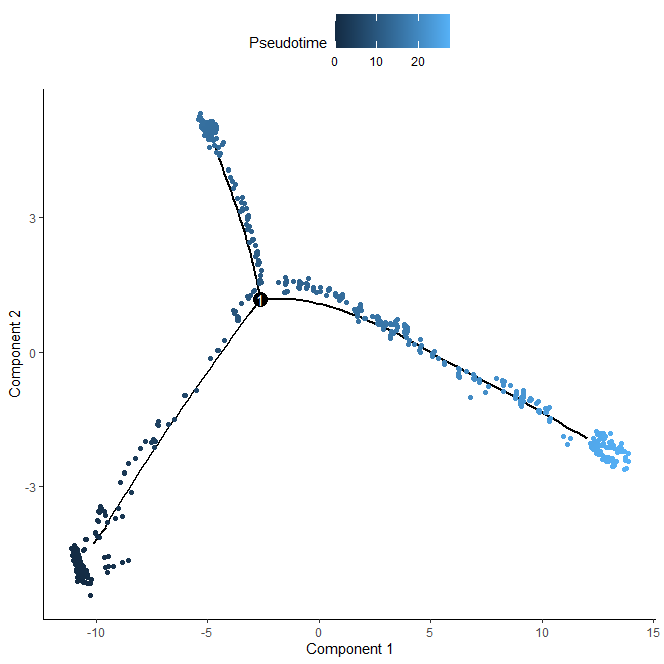


**Supplementary Figure 8.** Distribution of cells in each cluster on the pseudo-time trajectory.


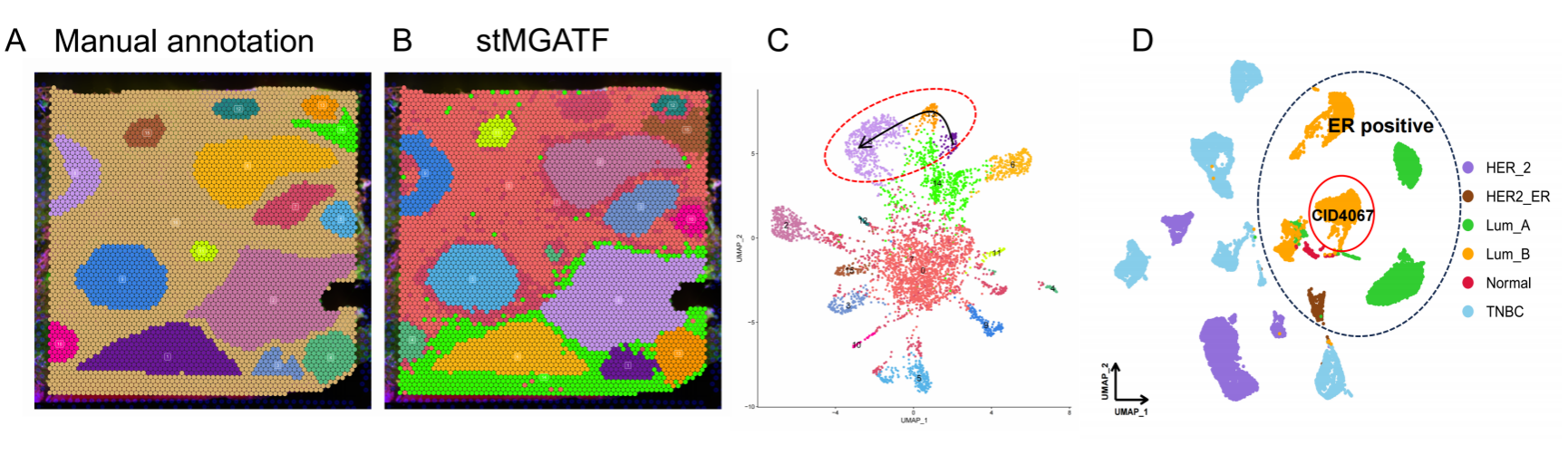


**Supplementary Figure 9.** Data analysis in breast cancer sample. **A.** Manual annotation. **B.** Spatial clustering by stMGATF. **C.** UMAP visualization of the latent features by stMGATF. **D.** UMAP visualization of 24,489 epithelial cells from 20 breast cancer patients.


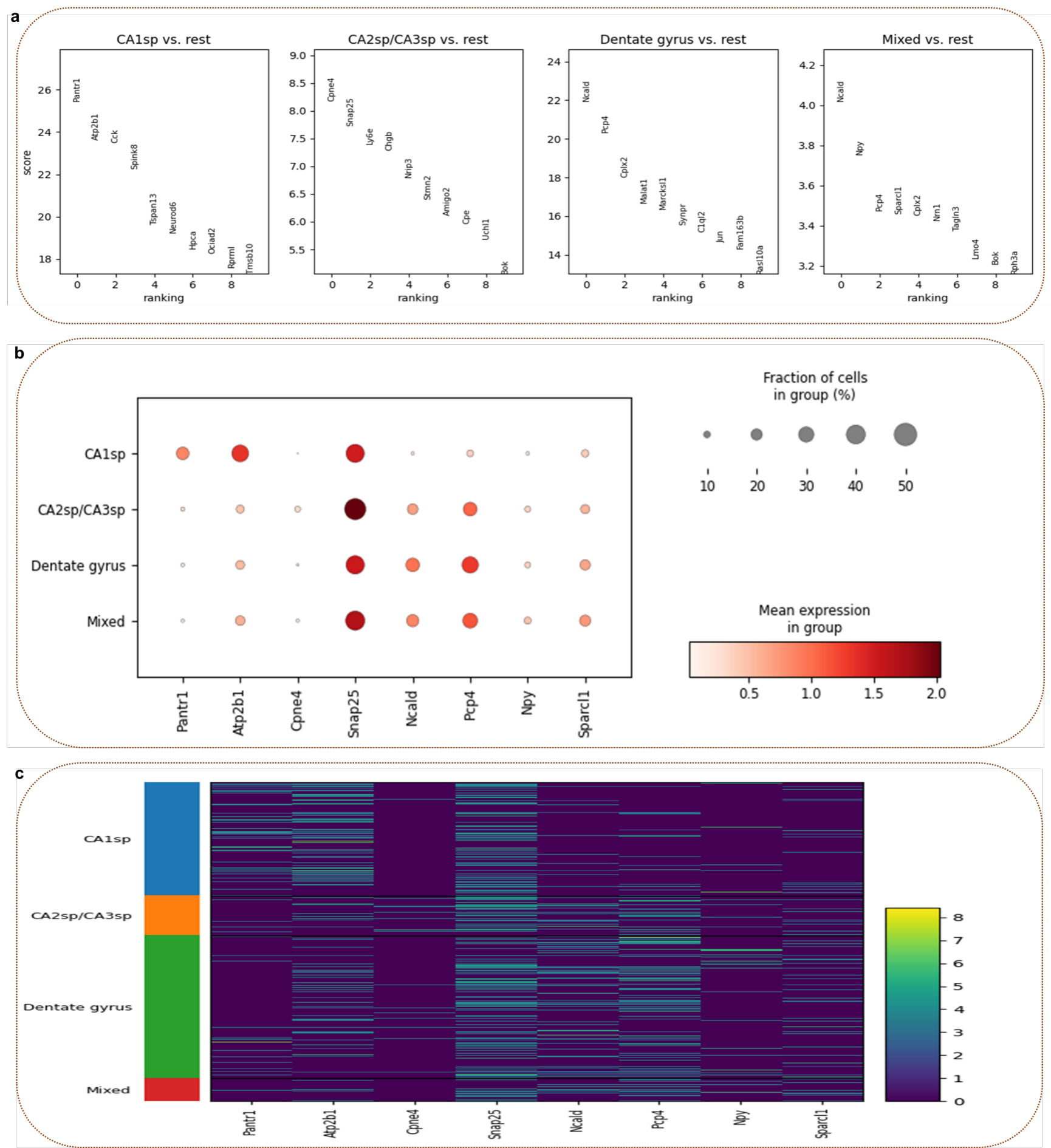


**Supplementary Figure 10.** stMGATF-2D uncovers the spatial domain-specific SVGs on the onto a pseudo-3D ST data constructed by aligning the spots of the "cord-like" structure in seven hippocampus sections profiled by Slide-seq. **a** Visualization of top-10 marker gene in each layer. **b** Dot map of top-2 marker gene in each layer. **c** Heatmap of top-2 marker gene in each layer.


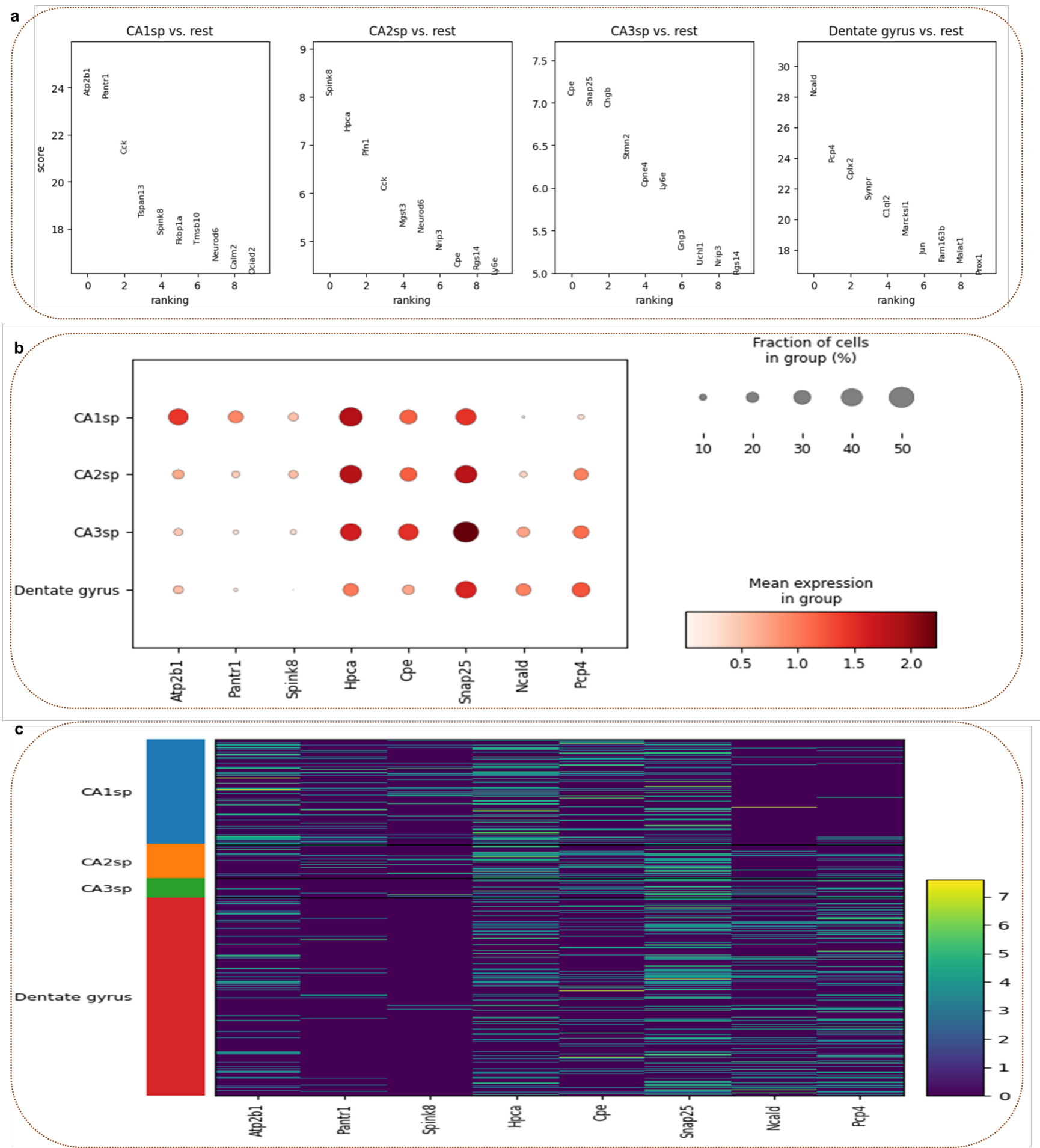


**Supplementary Figure 11.** stMGATF-3D uncovers the spatial domain-specific SVGs on the onto a pseudo-3D ST data constructed by aligning the spots of the "cord-like" structure in seven hippocampus sections profiled by Slide-seq. **a** Visualization of top-10 marker gene in each layer. **b** Dot map of top-2 marker gene in each layer. **c** Heatmap of top-2 marker gene in each layer.


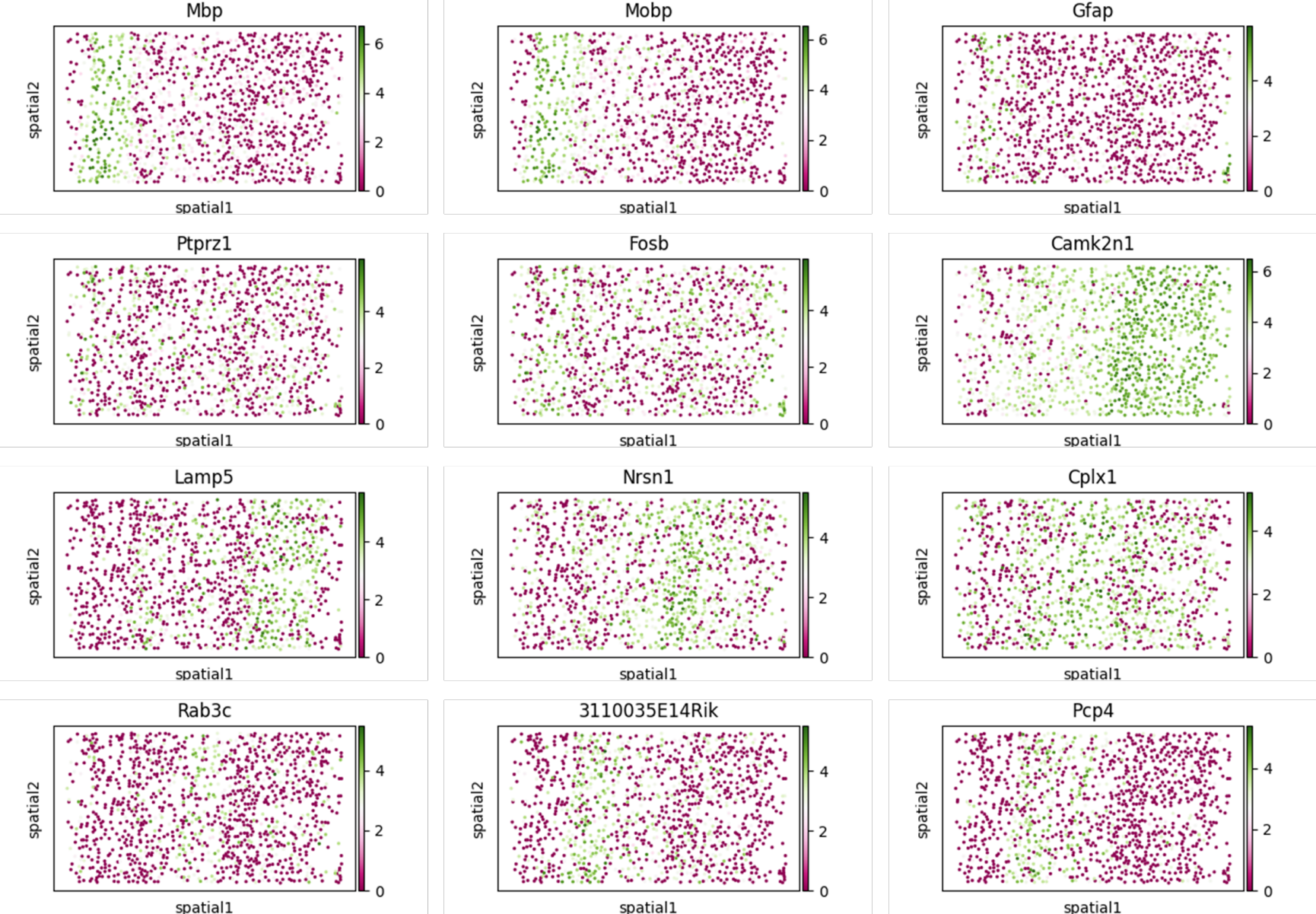


**Supplementary Figure 12.** Spatial expression patterns of SPVGs detected by stMGATF. *Mbp*, "*Mobp*" is more highly enriched in layer CC, *Gfap* and *Ptprz1* are more highly expressed in layer HPC, *Fosb*, *Camk2n1* is more highly enriched in layer1, *Camk2n1* and *Lamp5*" is more highly expressed in layer2/3, *Nrsn1* and *Camk2n1* is more highly enriched in layer4, *Cplx1* and *Rab3c* is more highly expressed in layer5, *Camk2n1*, "*3110035E14Rik*", "*Pcp4*" is more highly enriched in layer6.


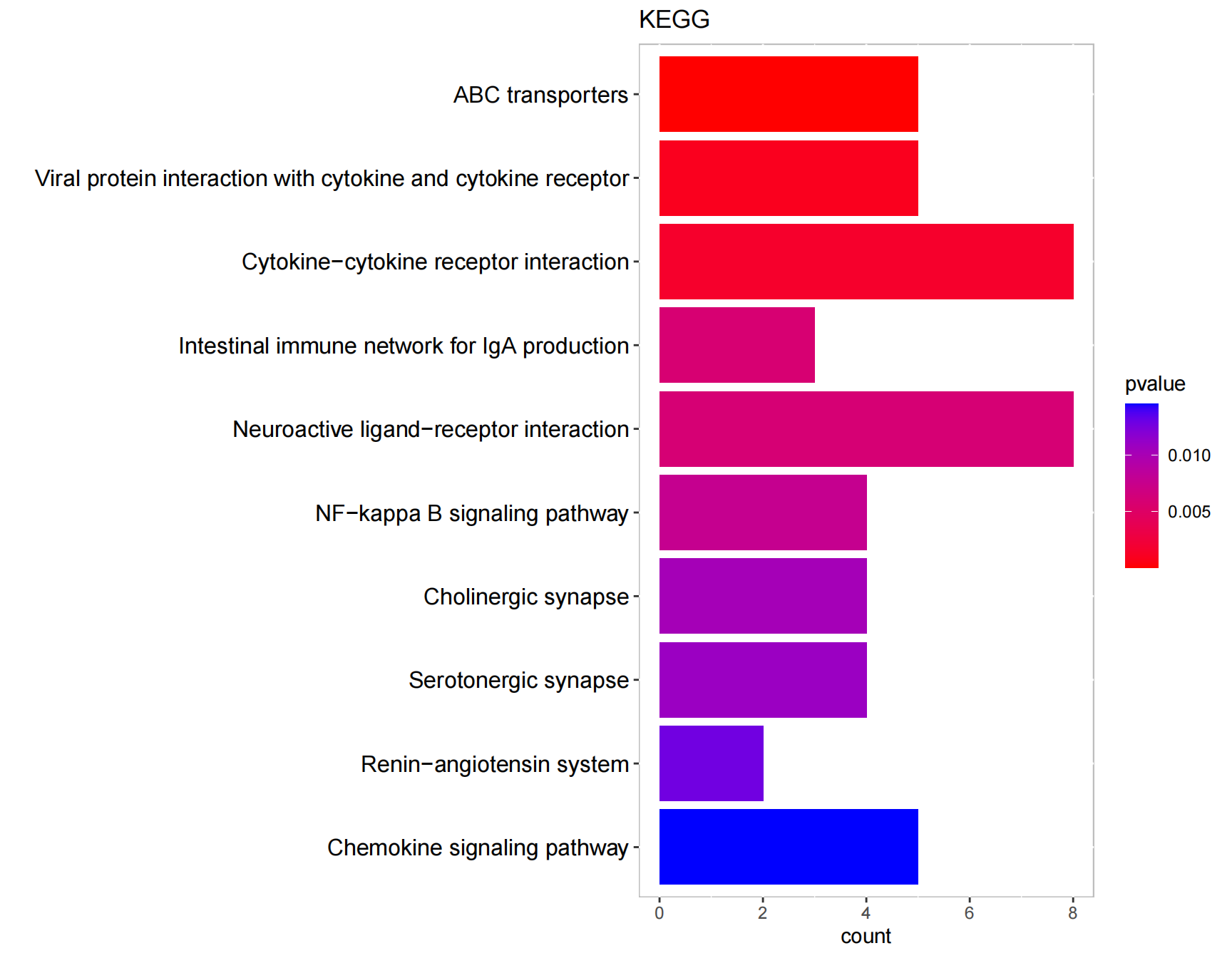


**Supplementary Figure 13.** KEGG functional enrichment analysis of spatial dark genes (SDGs). The significance threshold is 0.05.


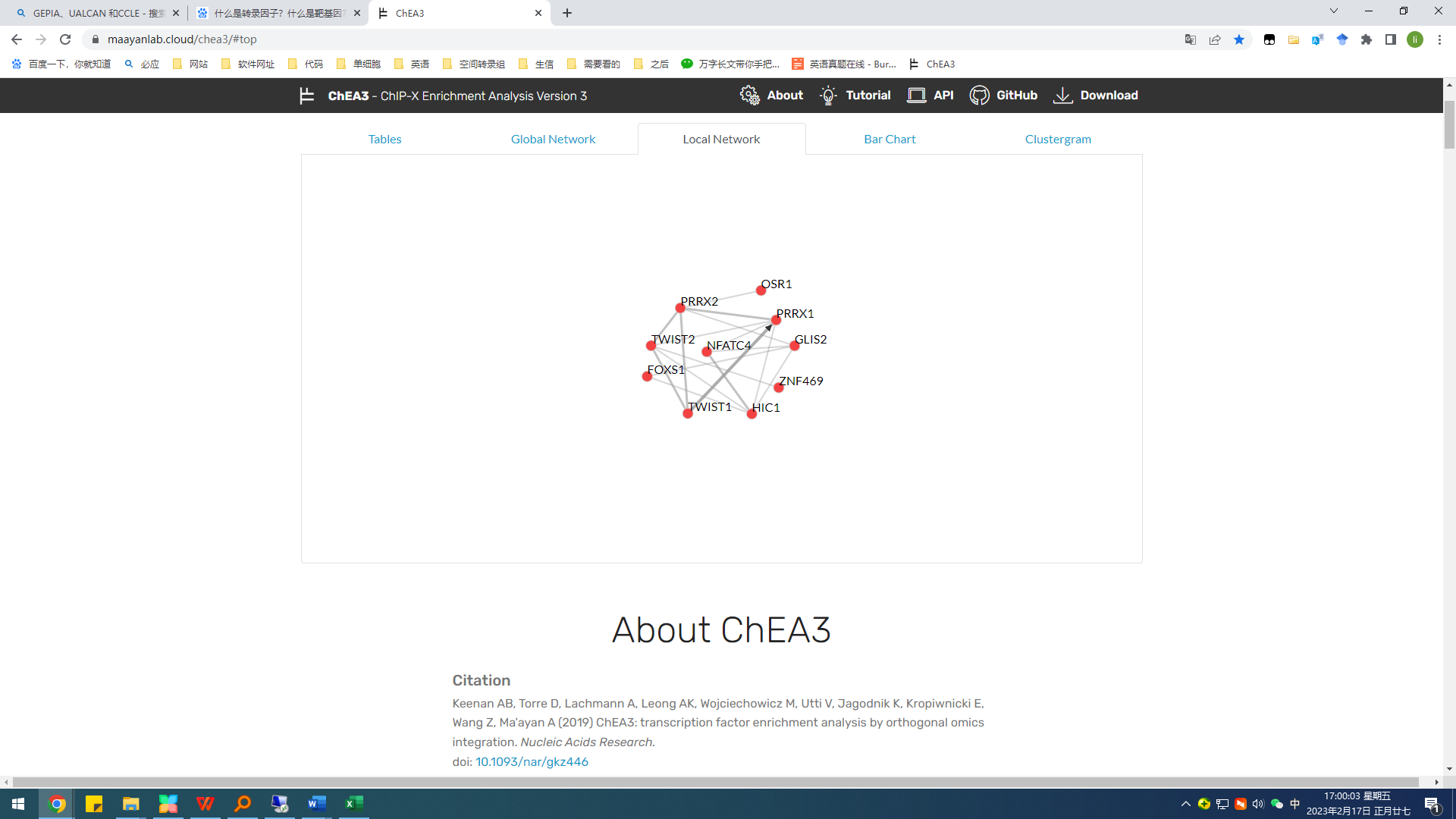


**Supplementary Figure 14.** Co-expression network of the top ten upstream transcription factors identified by SDGs.


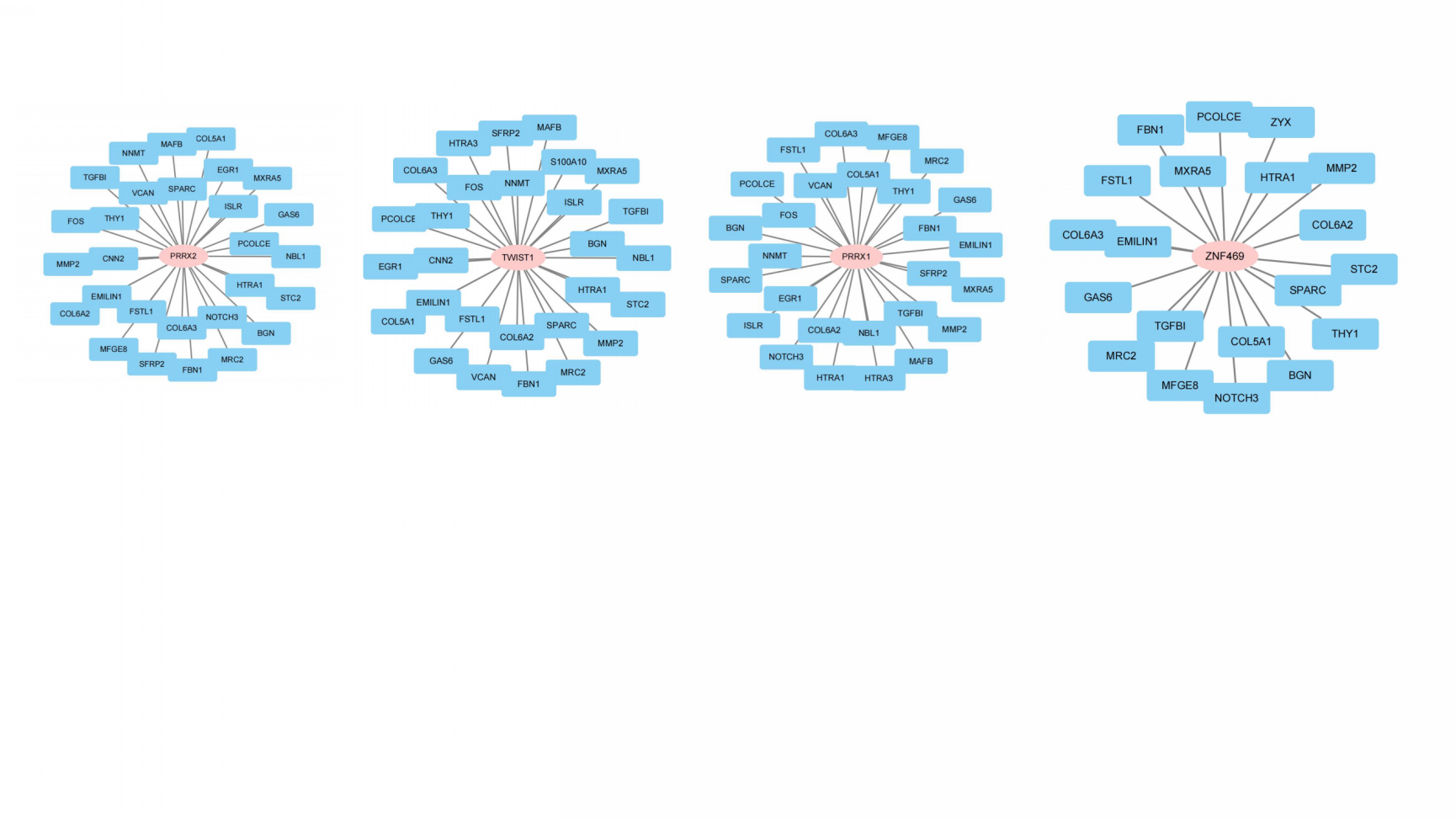


**Supplementary Figure 15.** Networks showed the relation of regulation from four ten upstream transcription factors to SDGs.


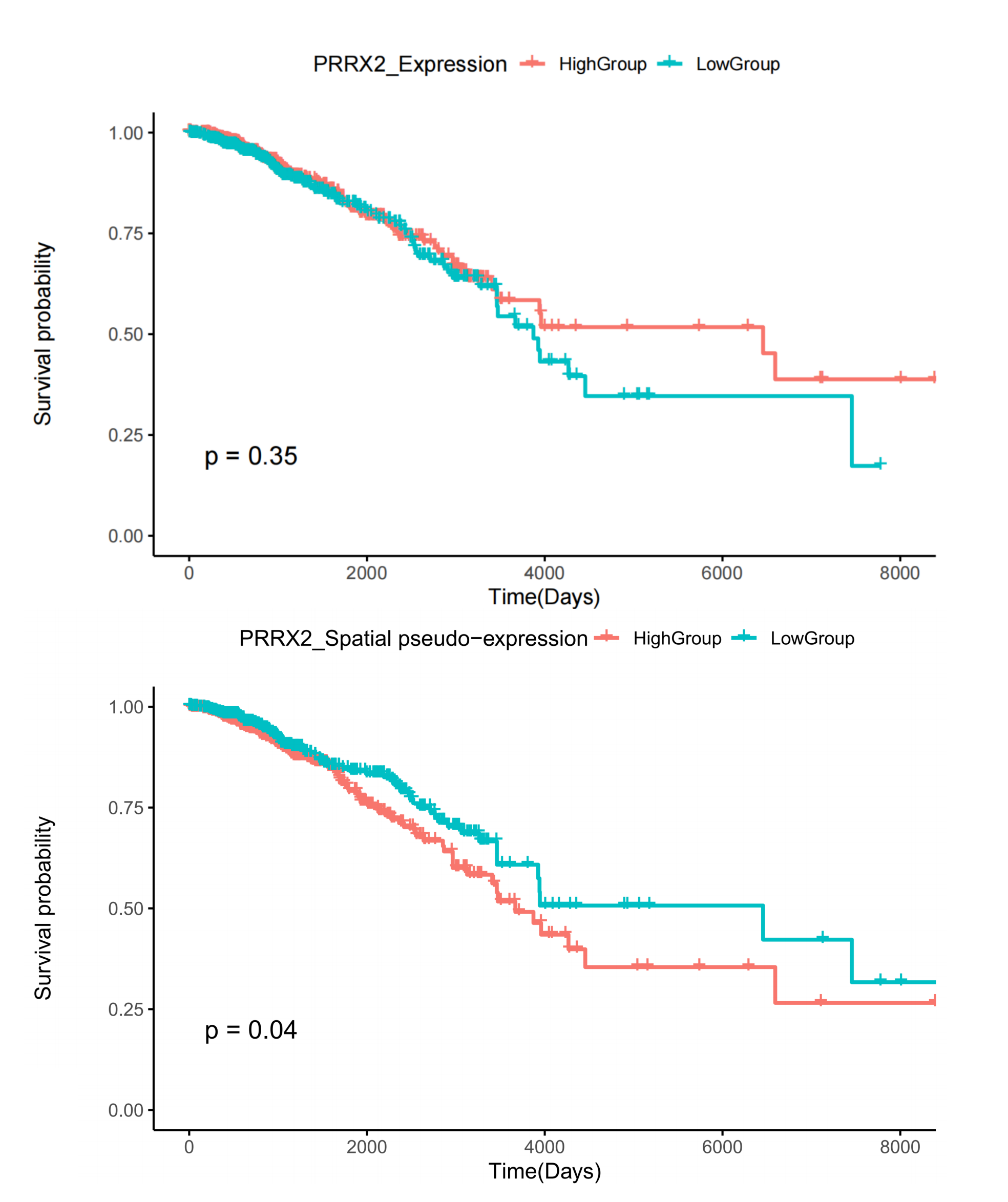


**Supplementary Figure 16.** Comparison of hub upstream TF PRRX2 survival curves in gene expression and network degree pseudo-expression constructed by stMGATF from RNA-seq data of breast cancer in TCGA database.


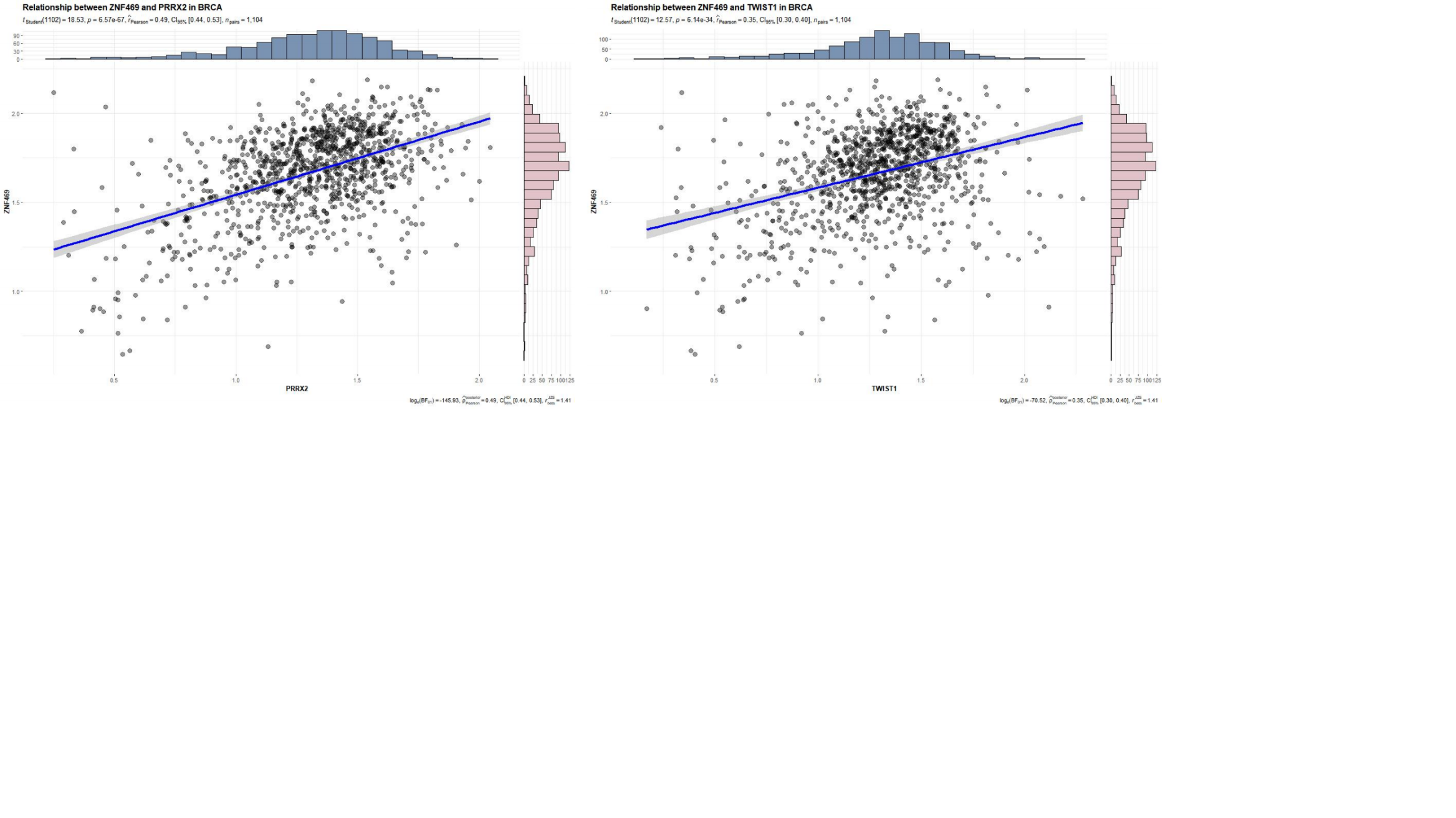


**Supplementary Figure 17.** Correlation scatter plot of ZNF469 and breast cancer metastasis related TFs expression in BRCA with lateral histogram.

**Supplementary Table 1.** The result of 10-fold cross-validation.

| K-fold=10 | ASW | ARI |
| --- | --- | --- |
| 1 | 0.4778 | 0.8229 |
| 2 | 0.4741 | 0.8501 |
| 3 | 0.4762 | 0.8489 |
| 4 | 0.4778 | 0.8500 |
| 5 | 0.4775 | 0.8505 |
| 6 | 0.4772 | 0.8499 |
| 7 | 0.4765 | 0.8494 |
| 8 | 0.4766 | 0.8509 |
| 9 | 0.4759 | 0.8509 |
| 10 | 0.4774 | 0.8503 |
| Average | 0.4767 | 0.8473 |

**Supplementary Table 2.** KEGG pathway analysis of SPVGs.

| KEGG |  |
| --- | --- |
| enriched biological process | Enriched p value |
| hsa04514:Cell adhesion molecules | 4.83E-13 |
| hsa04510:Focal adhesion | 1.10E-10 |
| hsa04512:ECM-receptor interaction | 3.47E-09 |
| hsa04060:Cytokine-cytokine receptor interaction | 9.82E-09 |
| hsa04062:Chemokine signaling pathway | 9.55E-08 |
| hsa04151:PI3K-Akt signaling pathway | 9.55E-08 |
| hsa04612:Antigen processing and presentation | 9.55E-08 |
| hsa04659:Th17 cell differentiation | 9.86E-08 |
| hsa05165:Human papillomavirus infection | 0.00000306 |
| hsa04974:Protein digestion and absorption | 0.000000293 |

**Supplementary Table 3.** GO enrichment analysis of transcription factors.

| Gene Ontology Consortium |  |
| --- | --- |
| enriched biological process | Enriched p value |
| extracellular region（GO:0005576） | 2.35069E-15 |
| extracellular matrix structural constituent（GO:0005201） | 4.63284E-13 |
| extracellular space（GO:0005615） | 3.06554E-11 |
| extracellular matrix（GO:0031012） | 9.07268E-08 |
| extracellular exosome（GO:0070062） | 7.40835E-07 |
| cell adhesion（GO:0007155） | 1.0746E-05 |
| integrin binding（GO:0005178） | 1.77658E-05 |
| extracellular matrix binding（GO:0050840） | 0.000437182 |
| extracellular matrix structural constituent conferring tensile strength（GO:0030020） | 0.000766341 |
| calcium ion binding（GO:0005509） | 0.005939981 |

References

1. Stuart, T. *et al.* Comprehensive Integration of Single-Cell Data. *Cell* **177**, 1888-1902.e21 (2019).

2. Zhao, E. *et al.* Spatial transcriptomics at subspot resolution with BayesSpace. *Nat Biotechnol* **39**, 1375–1384 (2021).

3. Dries, R. *et al.* Giotto: a toolbox for integrative analysis and visualization of spatial expression data. *Genome Biol* **22**, 78 (2021).

4. Bigness, J., Loinaz, X., Patel, S., Larschan, E. & Singh, R. Integrating Long-Range Regulatory Interactions to Predict Gene Expression Using Graph Convolutional Networks. *Journal of Computational Biology* **29**, 409–424 (2022).

5. Pham, D. *et al.* *stLearn: integrating spatial location, tissue morphology and gene expression to find cell types, cell-cell interactions and spatial trajectories within undissociated tissues*. http://biorxiv.org/lookup/doi/10.1101/2020.05.31.125658 (2020) doi:10.1101/2020.05.31.125658.

6. Dong, K. & Zhang, S. Deciphering spatial domains from spatially resolved transcriptomics with an adaptive graph attention auto-encoder. *Nat Commun* **13**, 1739 (2022).

7. Zuo, C. *et al.* Elucidating tumor heterogeneity from spatially resolved transcriptomics data by multi-view graph collaborative learning. *Nat Commun* **13**, 5962 (2022).

8. Maynard, K. R. *et al.* Transcriptome-scale spatial gene expression in the human dorsolateral prefrontal cortex. *Nat Neurosci* **24**, 425–436 (2021).

9. Rodriques, S. G. *et al.* Slide-seq: A scalable technology for measuring genome-wide expression at high spatial resolution. *Science* **363**, 1463–1467 (2019).

10. Rousseeuw, P. J. Silhouettes: A graphical aid to the interpretation and validation of cluster analysis. *Journal of Computational and Applied Mathematics* **20**, 53–65 (1987).
